# *Pax6* Is Required for Normal Cell-Cycle Exit and the Differentiation Kinetics of Retinal Progenitor Cells

**DOI:** 10.1371/journal.pone.0076489

**Published:** 2013-09-20

**Authors:** Chen Farhy, Michael Elgart, Zehavit Shapira, Varda Oron-Karni, Orly Yaron, Yotam Menuchin, Gideon Rechavi, Ruth Ashery-Padan

**Affiliations:** 1 Department of Human Molecular Genetics and Biochemistry, Sackler Faculty of Medicine and Sagol School of Neuroscience, Tel Aviv University, Tel Aviv, Israel; 2 Cancer Research Center, Chaim Sheba Medical Center, Tel Hashomer and Sackler Faculty of Medicine, Tel Aviv University, Tel Aviv, Israel; Telethon Institute of Genetics and Medicine, Italy

## Abstract

The coupling between cell-cycle exit and onset of differentiation is a common feature throughout the developing nervous system, but the mechanisms that link these processes are mostly unknown. Although the transcription factor *Pax6* has been implicated in both proliferation and differentiation of multiple regions within the central nervous system (CNS), its contribution to the transition between these successive states remains elusive. To gain insight into the role of *Pax6* during the transition from proliferating progenitors to differentiating precursors, we investigated cell-cycle and transcriptomic changes occurring in *Pax6* ^*-*^ retinal progenitor cells (RPCs). Our analyses revealed a unique cell-cycle phenotype of the *Pax6*-deficient RPCs, which included a reduced number of cells in the S phase, an increased number of cells exiting the cell cycle, and delayed differentiation kinetics of *Pax6* ^*-*^ precursors. These alterations were accompanied by coexpression of factors that promote (*Ccnd1*, *Ccnd2*, *Ccnd3*) and inhibit (*P27* ^*kip1*^ and *P27* ^*kip2*^) the cell cycle. Further characterization of the changes in transcription profile of the *Pax6*-deficient RPCs revealed abrogated expression of multiple factors which are known to be involved in regulating proliferation of RPCs, including the transcription factors *Vsx2*, *Nr2e1*, *Plagl1* and Hedgehog signaling. These findings provide novel insight into the molecular mechanism mediating the pleiotropic activity of *Pax6* in RPCs. The results further suggest that rather than conveying a linear effect on RPCs, such as promoting their proliferation and inhibiting their differentiation, *Pax6* regulates multiple transcriptional networks that function simultaneously, thereby conferring the capacity to proliferate, assume multiple cell fates and execute the differentiation program into retinal lineages.

## Introduction

During retinal development in vertebrates, a single pool of rapidly proliferating multipotent retinal progenitors cells (RPCs) gives rise to six different types of neurons and the Muller glia. Differentiation of all retinal cell types occurs in an evolutionarily conserved order and begins after terminal exit from the cell cycle [1-3]. Neurogenesis and progenitor proliferation occur simultaneously, thus at any given developmental stage some RPCs exit the cell cycle and differentiate while others continue to divide. The rate of proliferation and the fraction of RPCs that exit the cell cycle determine the size of the remaining progenitor pool as well as the number of each type of neuron generated. Thus, tight regulation of cell-cycle progression and exit is required to ensure the timely generation and correct amount of the various retinal cell types.

One of the greatest challenges in studying the regulation of RPC proliferation is the close coupling and interdependence of cell-cycle exit, cell-fate specification and neuronal differentiation. Thus, factors promoting neurogenesis or altering the cell fate acquired by ensuing postmitotic precursors may also directly regulate cell-cycle components or affect the timing of cell-cycle exit. For example, this dual activity was reported for the proneural gene *Neurog2*, which was found to promote neuronal differentiation in the spinal cord and at the same time suppress the expression of cell-cycle genes such as *Ccnd1* [4]. Similarly, *Ccnd1*, which promotes cell-cycle progression, was recently found to play a role in cell-fate acquisition and in establishing the correct proportion of the various retinal cell types [5].

The paired and homeodomain-containing transcription factor (TF) *Pax6* has been one of the most extensively investigated factors with respect to neural differentiation and progenitor proliferation. Its function has been studied in various animal models, in different areas of the CNS and at different stages of development. The roles of *Pax6* in retinogenesis have been investigated by systemic and *Cre*-mediated somatic mutations [6-9]. Deletion of *Pax6* from the peripheral optic cup (OC) revealed that the roles of *Pax6* within RPCs depend on their state of maturation: RPCs located in the distal OC are late to differentiate and require *Pax6* for inhibition of *Crx* and completion of neurogenesis. In contrast, RPCs located centrally, within the differentiation zone, require *Pax6* to retain their multipotency and differentiate into most retinal cell types, excluding subtypes of GABAergic amacrine cells [6,8].

In addition to its role in neuronal differentiation, *Pax6* is required for normal proliferation in the developing retina as well as in other regions of the CNS, including the telencephalon (reviewed in 10), diencephalon [11] and spinal cord [12]. In the optic vesicle (OV), as well as in the retina, *Pax6* is required to maintain high levels of proliferation as *Pax6* ^-^ progenitors display a significant reduction in bromodeoxy-uridine (BrdU) incorporation [6-8]. Nevertheless, it remains unclear whether these proliferative changes are due to a discrete role of *Pax6* in regulating RPC proliferation or are an indirect outcome of the altered cell fates observed in the various knockout models.

The aim of this study was to distinguish between *Pax6*’s role in cell-cycle dynamics and its activity in cell specification, and to elucidate the transcriptional network mediating its involvement in RPC proliferation. We therefore characterized the dynamics of the cell cycle and cell differentiation following conditional mutation of *Pax6* in RPCs, as well as the global changes in gene expression in the *Pax6*-mutant cells. These analyses revealed that the processes of cell-cycle exit, cell-fate specification and neuronal differentiation, which normally occur in close tandem succession, are temporally separated in *Pax6*-deficient RPCs, thus demonstrating a role for *Pax6* in both regulating progenitor proliferation and coupling cell-cycle exit and neuronal differentiation. Furthermore, our findings reveal that rather than conveying a positive linear effect on RPC proliferation, *Pax6* intersects with multiple retinal programs, some of which promote RPC proliferation, while others promote cell-cycle exit and differentiation.

## Materials and Methods

### Mouse Lines

The *Pax6* ^*flox*^ allele contains *loxP*s flanking the initiator ATG and exons 4–6 encoding the paired domain [13]. The α*-Cre*-transgenic line contains the *Pax6* P0 promoter and the peripheral retina enhancer (termed α) followed by *Cre* which was cloned 5′ of *IRES-intron-gfp-pA* [6]. All animal work was conducted according to national and international guidelines and all efforts were made to minimize suffering. The protocol was approved by the Tel Aviv University institutional animal care and use committee (IACUC permit: M08092).

### Immunofluorescence and In Situ Hybridization (ISH)

Immunofluorescence analysis was performed as described previously [8]. The primary antibodies used are shown in Table S3. Secondary antibodies were conjugated to Alexa488, Alex594 (1:1,000, Invitrogen) or Aminomethylcoumarin Acetate (AMCA) (1:100, Jackson Immuno Research Laboratories).

ISH analysis was performed on frozen sections. Hybridization was conducted overnight at 65°C and performed as described previously [8]. Probes used for ISH were: *FoxN4* (king gift of Xiang Ming [14]), *Nr2e1* (amplified from cDNA using tcctgaacggcagactctcg and cgaggttgcctgacctacgg cloned into *pGEM-T-easy*, Promega), *Plagl1* (king gift of Carol Schuurmans [15]), and *Gli1, Gli2, Gli3* (king gift of Alexandra Joyer).

Slides were viewed with an Olympus BX61 fluorescent microscope or laser-scanning confocal microscope CLSM 410 (Zeiss) and images were analyzed using the image analysis system 'AnalySIS'.

### Analysis of Cell-Cycle Exit

To label proliferating cells, 5-bromo-2-deoxyuridine (BrdU, 140 µg/g body weight, Sigma) was injected intraperitoneally (IP) into pregnant females at the desired developmental stage. To assay for cell-cycle exit, BrdU was injected 24 h before embryo harvesting. Tissues were then processed for embedding in paraffin and sectioned. Sections were stained for BrdU incorporation and either proliferating cell nuclear antigen (PCNA) or the proliferating cell marker Ki67 using immunofluorescence. No less than three central sections from three different eyes were counted for both BrdU^+^ and BrdU ^+^ Marker^+^. Cell-cycle exit index was calculated by dividing the total number of BrdU ^+^ Marker^+^ cells scored in all sections of the same eye by the total number of BrdU^+^ cells scored in all sections of the same eye. All of the values for a single developmental stage and genotype were averaged and standard deviation (SD) was calculated. Values obtained for control and *Pax6*-mutant animals were compared using either Student’s t-test or Mann–Whitney test to determine statistical significance.

### Expression Microarray

The control and mutant *Pax6* ^*-*^ RPCs were isolated by fluorescent-activated cell sorting (FACS) from *Pax6* ^*+/+*^;α*-Cre* or *Pax6* ^*loxP/loxP*^;α*-Cre* E12 (embryonic day 12) eyes. Three pools of 1.8 x 10^6^ cells each were collected for the analyses. RNA was isolated using TRIZOL reagent. Hybridization was to Affymetrix Mouse 430.2 gene-expression arrays according to the manufacturer’s protocol. For microarray data processing, we used remote analysis computation for gene expression data (RACE [16]) front-end for the Bioconductor [17] package of the R language utilizing the RMA (Robust Multichip Average; [18]) algorithm. Results were filtered by fold-change (1.5), *p*-value (0.05), and FDR (false discovery rate, 10%). The results were further verified using SAM (Significance Analysis of Microarrays) software [19]. The expression data were submitted to the NCBI Gene Expression Omnibus (http://www.ncbi.nlm.nih.gov/geo) under series accession no. GSE45143.

## Results

### Delayed Neuronal Differentiation in the *Pax6*-Deficient Retina

**Figure 1:**
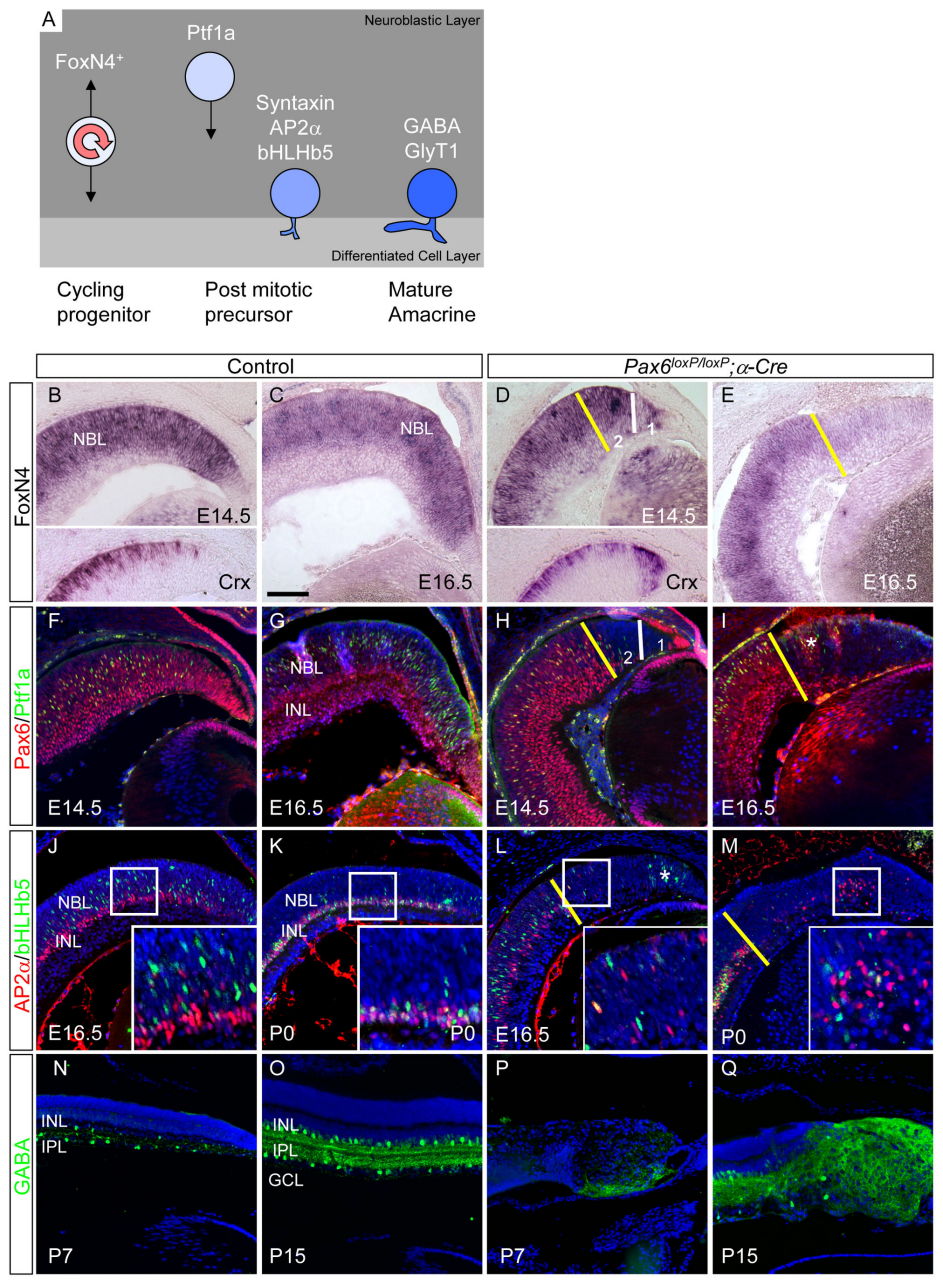
Delayed differentiation of amacrine precursors in the *Pax6* ^*loxP/loxP*^ ;α*-Cre* retina. (A) A scheme of the stages of amacrine interneuron differentiation. Amacrine cells evolve from FoxN4-expressing RPCs. In the postmitotic amacrine precursors, FoxN4 is reduced and Ptf1a expression is initiated. The Ptf1a-positive precursors migrate to the prospective INL, lose Ptf1a expression and initiate expression of TFs involved in the differentiation of amacrine subtypes (e.g. Ap2α and bHLHb5). The final differentiation of amacrine cells occurs a few days after birth with accumulation of neurotransmitters and transporters (e.g. GABA, glycine transporter GlyT1). Expression of amacrine specification and differentiation markers in control (B,C,F,G,J,K,N,O) and *Pax6^loxP/loxP^*;*α-Cre* OC (D,E,H,I,L,M,P,Q). Expression of FoxN4 (B–E, the inset in B and D is Crx on adjacent section) as detected using ISH. Indirect immunofluorescence (IIF) was employed to detect the expression of Ptf1a and Pax6 (green and red, respectively, F–I; adjacent sections to B-E respectively), bHLHb5 and Ap2α (green and red, respectively, J–M) and GABA (green, N–Q) during various stages of development as indicated. Pax6 (red in F–I and not shown) and Crx (inset in B,D and not shown) expression determined by IIF or ISH was used to identify the Pax6-deficient area (yellow line in D,E,H,I,L,M) and to delineate the neurogenic and nonneurogenic RPC populations in the *Pax6* ^loxP/loxP^;α*-Cre* retina (numbered 1 and 2 and separated by a dotted white line in D and H). Abbreviations: GCL, ganglion cell layer; INL, inner nuclear layer; IPL, inner plexiform layer; NBL, neuroblastic layer. Scale bar in C is 100 µm.

Previous studies of systemic and conditional *Pax6* deletions have shown both reduced proliferation and limited differentiation of the *Pax6*-deficient RPCs exclusively to the GABAergic (producing γ-aminobutyric acid) amacrine lineage [6-8]. To distinguish the proliferative roles of *Pax6* from its effects on cell-fate acquisition, we characterized the dynamics of differentiation into amacrine interneurons in *Pax6* conditional knockout (*Pax6* ^*loxP/loxP*^;α*-Cre*) and control mice. We determined the temporal and spatial expression patterns of genes that are known to participate in the regulation of amacrine cell differentiation based on gene-expression studies of mutant mice (Figure 1A). The TF FoxN4 is an upstream regulator of amacrine and horizontal lineages [14]. *FoxN4* is expressed by proliferating RPCs located in the neuroblastic layer (NBL) of the embryonic retina (E14.5, E16.5, Figure 1B,C and [14]). In the *Pax6* ^*loxP/loxP*^;α*-Cre* OC (Figure 1D,E), we defined distal and proximal *Pax6* ^-^ populations by detection of *Crx* and *Pax6* using indirect immunofluorescence (IIF) analysis on adjacent sections (*Crx* – inset in Figure 1B,D; *Pax6* – Figure 1H,I). We found that in the *Pax6*-mutant region, *FoxN4* is initially retained in *Pax6* ^-^ *Crx* ^*-*^ neurogenic progenitors (Figure 1D area 2) and only shows a decrease in distally located *Pax6* ^-^ *Crx* ^*+*^ cells (Figure 1D area 1). However, at E16.5, *FoxN4* expression was lost from virtually all *Pax6* ^-^ cells (Figure 1E).

As *FoxN4* is downregulated once cells exit the cell cycle [14], the reduced number of *FoxN4*-expressing cells may reflect accumulation of postmitotic amacrine precursors. We therefore examined the expression of TFs known to be expressed in these precursors and to be required for generation of amacrine subtypes. *Ptf1a* is transiently expressed in postmitotic amacrine and horizontal precursors located in the NBL (Figure 1F,G, green and [20,21]). Once *Ptf1a* expression is extinguished, amacrine precursors start to express *Bhlhb5* and *AP2a* (Figure 1J,K, green and red respectively). These are first detected in migrating precursors in the NBL (E16.5, Figure 1J and [22,23]) and are later restricted to the prospective inner nuclear layer (INL, P0, Figure 1K).

Surprisingly, although *Pax6*-deficient RPCs expressed *FoxN4* at E14, correlating with their competence to differentiate to amacrine cells, the dynamics of amacrine differentiation was perturbed in the *Pax6* ^-^ retina based on the reduced number of cells expressing *Ptf1a* at E14.5 and E16.5 in both *Pax6* ^-^ *Crx* ^*-*^ and *Pax6* ^-^ *Crx* ^*+*^ areas of the *Pax6* ^*loxP/loxP*^;α*-Cre* retina (Figure 1H,I). Reduced *Ptf1a* expression was further accompanied by a similar reduction in the expression of other amacrine precursor markers such as *bHLHb5*, *Ap2α*, *Ap2β*, *VC1.1*, *BarHL2* and *syntaxin* (E16.5, Figure 1L,M and Figure S1). However, despite reduced expression of amacrine precursor genes at embryonic stages, by P0 expression of some of the amacrine TFs, such as *Ap2α* (Figure 1M, red), was detected in an increasing number of *Pax6* ^-^ cells, while expression of other markers, such as *bHLHb5*, remained low (Figure 1M green). Taken together, these results demonstrate that while *Pax6* is not required to convey amacrine competence in RPCs, it is needed for the correct timing of expression and full repertoire of TFs involved in differentiation of the amacrine subtypes.

Considering the delayed onset of expression of amacrine precursor genes, we next monitored the accumulation of GABA (Figure 1N-Q), an inhibitory neurotransmitter which is detected in most *Pax6*-deficient amacrine interneurons in the *Pax6* ^*loxP/loxP*^ *; α-Cre* retina (Figure 1Q and [6]). Normally, GABA is first detected in the mouse retina at around birth [24]. However, in *Pax6* ^-^ amacrines, GABA accumulation was delayed and typically appeared only around P7 (Figure 1P,Q). Nevertheless, by P15, all *Pax6* ^-^ cells expressed GABA (Figure 1Q). Thus, despite reduced proliferation of *Pax6* ^-^ RPCs, the differentiation of *Pax6*-mutant cells to the GABAergic amacrine lineages is delayed, revealing a role for *Pax6* in the timing of neuronal differentiation.

### Pax6 Regulates the Timing of Cell-Cycle Exit of RPCs

**Figure 2:**
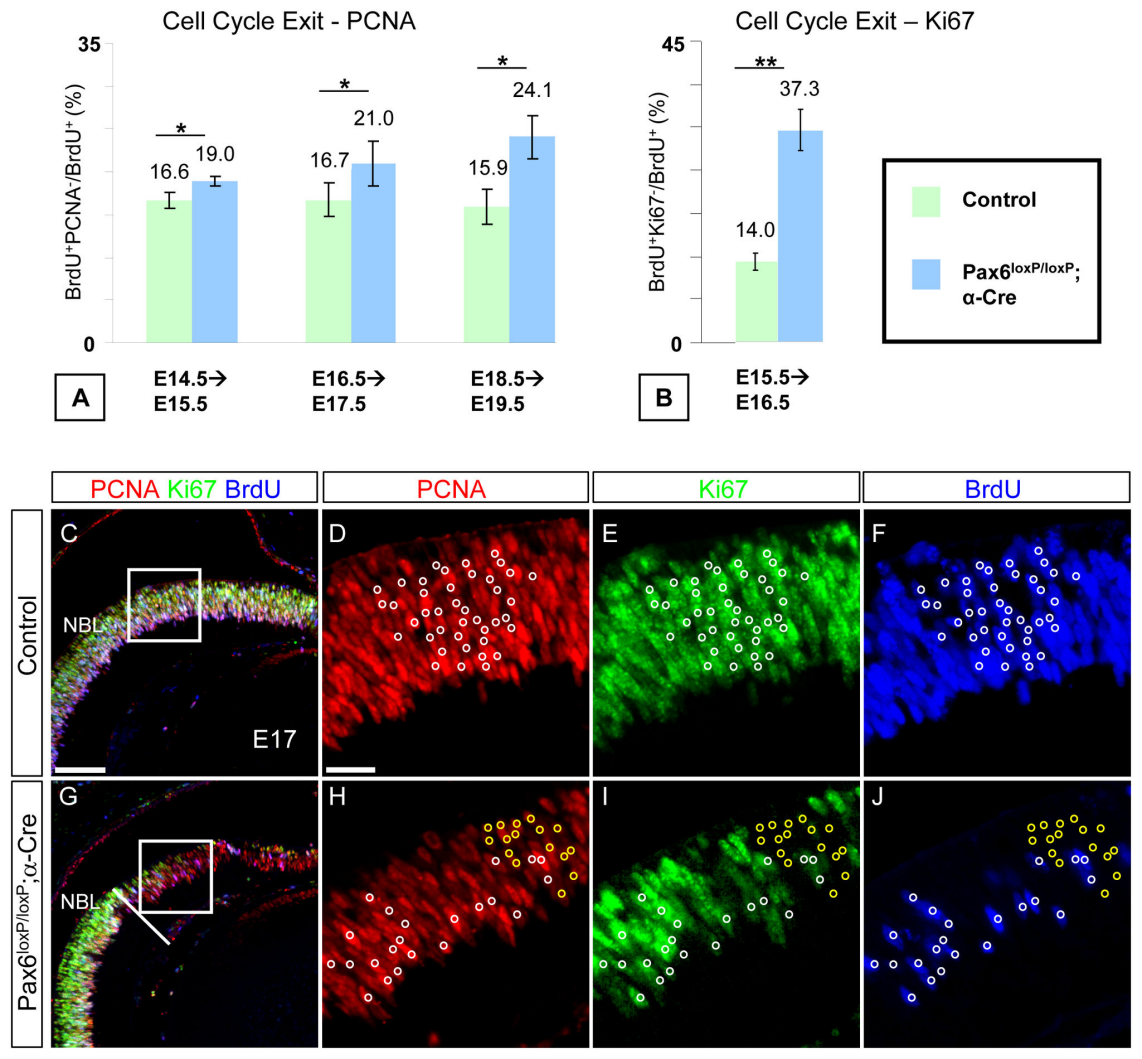
Neurogenic Pax6^-^ RPCs in the *Pax6* ^*loxP/loxP*^ ;α*-Cre* retina display increased cell-cycle exit and sustained expression of cell-cycle factors. A single pulse of BrdU was administered at E14.5, E15.5, E16.5 and E18.5, 24 h prior to sacrifice as indicated (A, B). Pax6 expression was detected on adjacent sections to identify the recombination area in the *Pax6^loxP/loxP^*;α*-Cre* OC. (A) The percentage of BrdU ^+^ PCNA^-^/BrdU^+^_Total_ was determined for the Pax6^*loxP/loxP*^ control (green bars) and *Pax6* ^*loxP/loxP*^ ;α*-Cre* (blue bars) distal retina. *Pax6* ^-^ RPCs show increased cell-cycle exit at all stages tested (19% (SD=0.6%), 21% (SD=2.7%) and 24.1% (SD=2.6%) compared to 16.6% (SD=0.93%), 16.7% (SD=2%) and 15.9% (SD=2%) in control at E14.5, E16.5 and E18.5, respectively; *p*≤0.05, n≥3). (B) The number of BrdU^+^ and BrdU ^+^ Ki67^-^ cells was quantified and the ratio BrdU ^+^ Ki67^-^/BrdU^+^_Total_ was used to compare cell-cycle exit rate in control (green bar) and *Pax6* ^*loxP/loxP*^ ;α*-Cre* (blue bar) distal retina at E15.5. *Pax6* ^-^ RPCs show increased cell-cycle exit (37.3% (SD=3.6%) in *Pax6* ^-^ compared to 14% (SD=1.5%) in control; *p*<0.01, n=6 for both genotypes). Triple immunofluorescence for PCNA, Ki67 and BrdU (red, green and blue, respectively, in C–J) in control (C–F) and *Pax6* ^*loxP/loxP*^ ;*α-Cre* (G–J) distal retina showing mitotic PCNA ^+^ Ki67 ^+^ BrdU^+^ in both control and mutant (white circles in D–F,H–J) and abnormal PCNA ^+^ Ki67^-^BrdU^-^ cells detected only in *Pax6* ^*loxP/loxP*^ ;α*-Cre* (yellow circles in H–J) OC. Scale bars in C,D are 100 and 25 µm, respectively.

The reduced proliferation previously documented in the *Pax6*-mutant retina [8] and the delayed differentiation based on reduced expression of amacrine differentiation genes (*Ptf1a*, Figure 1) detected in the *Pax6* ^-^ OC implicate altered cell-cycle kinetics. To investigate the cell-cycle kinetics of *Pax6* ^-^ RPCs, we compared the number of cells exiting the cell cycle in control and *Pax6* ^*loxP/loxP*^;α*-Cre* distal retinas. Cycling cells were labeled with a pulse of BrdU 24 h prior to sacrifice followed by detection of PCNA and BrdU by double IIF [5]. Total BrdU-positive cells as well as PCNA-negative BrdU-positive (PCNA^-^BrdU^+^) cells were counted and postmitotic cell index was calculated as PCNA^*-*^BrdU ^+^ /BrdU^+^_(Total)_ in control and *Pax6* ^*loxP/loxP*^;α*-Cre* retinas (Figure 2, Figure S2). This index reports the total number of postmitotic RPCs generated during a 24-h period following BrdU administration.

In the control retina, cell-cycle exit index was 16.6 ± 0.93%, 16.7 ± 2% and 15.9 ± 2% at E14.5, E16.5 and E18.5, respectively (green bars in Figure 2A). A significant (*P* ≤ 0.05) increase in the cell-cycle exit index was documented in the *Pax6* ^*loxP/loxP*^;α*-Cre* OC at all time points tested: 19 ± 0.6%, 21 ± 2.7% and 24.1 ± 2.6% at E14.5E15.5, E16.5E17.5, E18.5E19.5/P0, respectively (blue bars in Figure 2A). Thus, despite the obvious delay in differentiation of *Pax6* ^-^ RPCs and in agreement with reduced proliferation, there is a significant increase in cell-cycle exit of the *Pax6*-deficient RPCs as compared to controls and this increase becomes more prominent during the course of retinogenesis (1.14-, 1.25- and 1.51-fold at E14.5, E16.5 and E18.5, respectively).

In an attempt to reconcile the seemingly contrasting findings of increased cell-cycle exit but delayed differentiation, we tested control and *Pax6* ^*loxP/loxP*^;α*-Cre* retinas for expression of additional proliferating-cell markers. Ki67 has been previously shown to be expressed in proliferating cells and is downregulated in quiescent and differentiated cells [25-27]. In the retina, Ki67 expression fully overlapped with PCNA expression in proliferating cells (Figure 2C-E and [28]); however, postmitotic cells were previously reported to extinguish Ki67 prior to downregulating PCNA expression [29]. Surprisingly, we found progressive accumulation of PCNA ^+^ Ki67^-^ cells in the distal retina of *Pax6* ^*loxP/loxP*^ *; α-Cre* mice. These were initially detected at E14.5 and gradually increased, until by P0 most PCNA^+^ cells were Ki67^-^ (Figure 2G–I and Figure S2J-L). To determine the proliferative potential of the abnormal PCNA ^+^ Ki67^-^ progenitors, we tested their ability to incorporate BrdU during a 1.5-h pulse of BrdU administered at E17.5. As expected, in the control embryos, all PCNA^+^ cells were also Ki67^+^ and some of these were also BrdU^+^ (white circles in Figure 2D–F). In the *Pax6* ^-^ retina, BrdU incorporation was detected only in Ki67^+^ cells (white circles in Figure 2H–J) but BrdU was not incorporated into the PCNA ^+^ Ki67^-^ population (yellow circles in Figure 2H–J). To compare accumulation of normal (PCNA^-^Ki67^-^) and aberrant (PCNA ^+^ Ki67^-^) postmitotic cells, the cell-cycle index was recalculated using Ki67 instead of PCNA. This analysis, conducted from E15.5 to E16.5, revealed a 2.67-fold increase in the number of cells that exited the cell cycle in *Pax6* ^*loxP/loxP*^;α*-Cre* mice, as 37.3% (SD=3.6%) of the proliferating cells that incorporated BrdU at E15.5 were Ki67 negative by E16.5 compared to only14% (SD=1.5%) in the control (*P*≤0.001) (Figure 2B). Consistent with reduction in the number of Ki67-expressing cells, we observed a reduced number of cells expressing cyclin B1 (Ccnb1), which in the NBL is located in phase G2/M cells (Figure S2M,N [28]).

These results demonstrate a dramatic increase in the production of postmitotic cells in the *Pax6* ^-^ retina and reveal, for the first time, that in the *Pax6* ^*loxP/loxP*^;α*-Cre* retina, there is accumulation of an abnormal population of postmitotic PCNA ^+^ Ki67^-^ cells. These cells inhabit the NBL and seem to maintain neuroepithelial morphology until late stages of retinogenesis. *Pax6* is therefore required to maintain progenitors in the cell cycle as well as for the downregulation of cell-cycle factors, such as PCNA and Ccnd1 (see below) in the postmitotic precursors.

### High-Throughput Transcriptome Analysis of Pax6^-^ RPCs Reveals Altered Expression of Genes Associated with Cell Proliferation

**Figure 3:**
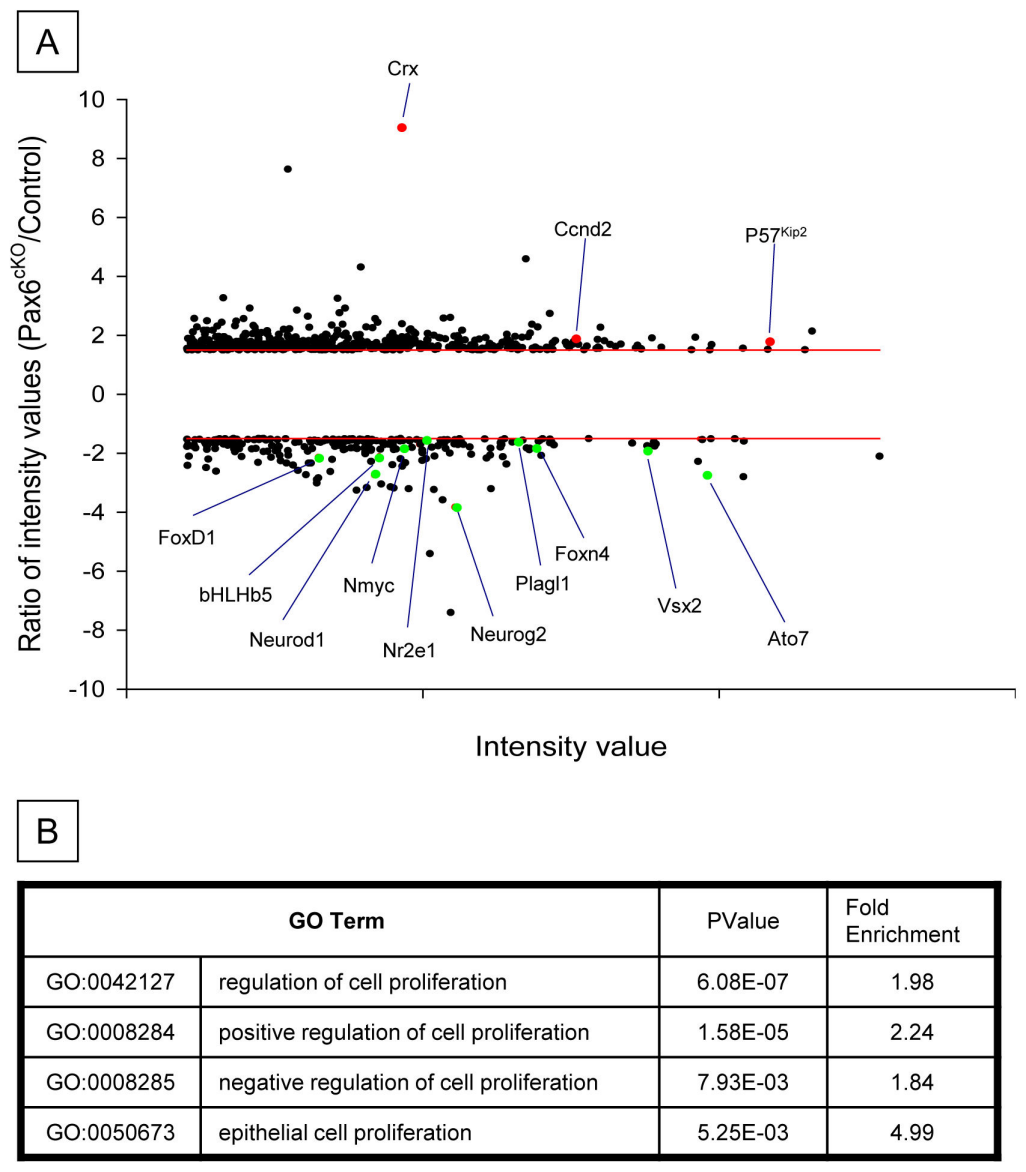
High-throughput analysis of transcriptional alterations in Pax6^-^ RPCs. (A) Scatterplot representing the fold change in gene expression in *Pax6* ^-^ versus control retinas (y axis) plotted against the intensity value in the control (x axis). Each spot corresponds to one gene; only genes whose expression changed at least 1.5-fold are shown. (B) Gene ontology (GO) analysis conducted using DAVID bioinformatics resource [32] for genes whose expression was altered in *Pax6* ^-^ versus control retinas, showing enrichment in various proliferation- and cell-cycle-related GO terms.

To identify the gene network operating downstream of *Pax6* to regulate cell proliferation and fate, we determined the transcriptomic changes in *Pax6*-deficient RPCs using microarray analysis. The expression profile was examined in E12 retina when both the proliferative and cell-cycle-related phenotypes are first detected and prior to onset of cell differentiation. *Pax6*-deficient and control RPCs were collected by FACS from mutant (*Pax6* ^*loxP/loxP*^;α*-Cre*) and control (*Pax6* ^*+/+*^ *α-Cre*) E12.5 retinas. This was possible because GFP is expressed in conjunction with Cre from the α*-Cre* construct [6]. Three biological replicates were conducted for each of the two genotypes and the gene-expression profiles were compared using Affymetrix Gene Chip microarray (MOE430-2). The expression of 952 genes was found to be altered between control and mutant RPCs (fold-change >1.5, *P*<0.05, FDR<10%); of these, 316 were downregulated and 636 were upregulated (Figure 3A and Table S1). Expression of *Pax6* was reduced threefold, as was the expression of the previously reported retinal *Pax6* targets *Atoh7*, *NeuroD1*, *Neurog2*, *FoxG1*, *FoxD1*, *delta-catenin* (*Ctnnd2*) and *Crx* (Figure 3A and [6,8,30,31]). Altered expression of over 20 genes found to be differentially in the microarray was validated using either IF or in situ hybridization (Table S2). Furthermore, testing the list of altered genes for enriched gene ontology (GO) terms using DAVID Bioinformatics Resources [32,33] and clustering significantly enriched (*P*<0.05) terms into functional groups indicated that these genes have known roles in previously reported *Pax6* functions such as eye development, cellular adhesion [34-36], neurogenesis and proliferation (Figure 3B and Figure S3). Therefore, the microarray data faithfully represented the molecular phenotype of the *Pax6*-mutant RPCs and reveals significant changes in expression of genes associated with cell proliferation.

### Pax6^-^ RPCs Overexpress Proteins Involved in both Cell-Cycle Withdrawal and Progression

The RPC decision to either differentiate or continue proliferating occurs during the G1 phase of the cell cycle. At this stage, proliferation can be promoted by the phosphorylation of *Rb* by a complex of D/E cyclins and cyclin-dependent kinases (CDKs). This phosphorylation is inhibited by CDK inhibitors (CKIs), which function to promote cell-cycle exit [5,37,38]. Altered expression of cell-cycle-related factors has been documented in the developing cortex and spinal cord of *Pax6* mutants [39-41].

**Figure 4:**
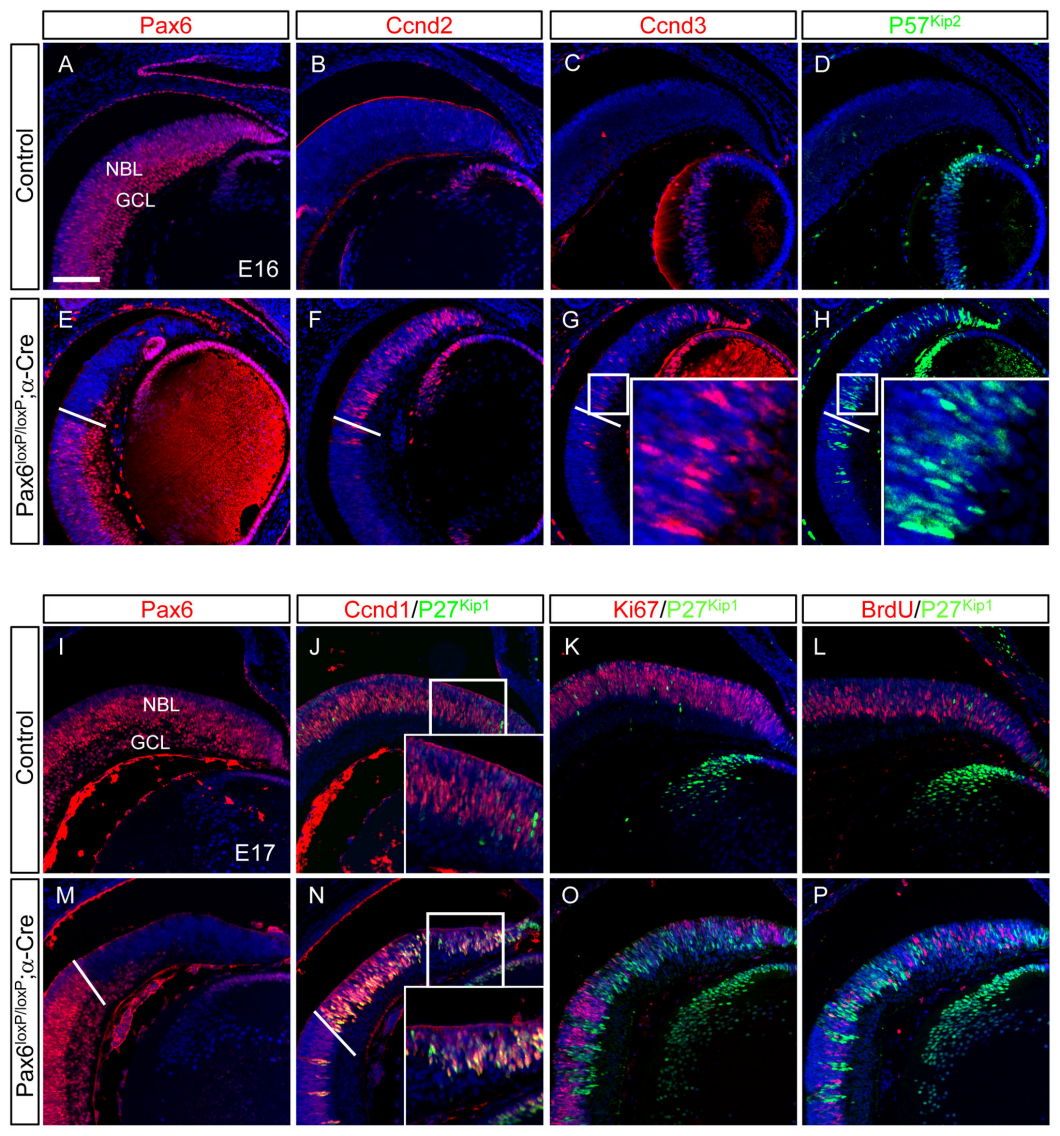
Increased expression of cell-cycle factors in the *Pax6* ^*loxP/loxP*^ ;α*-Cre* retina. Expression of Pax6 (A,E,I,M) and of cell-cycle progression and withdrawal factors was monitored using immunofluorescence in control (A–D,I–L) and *Pax6* ^*loxP/loxP*^ ;α*-Cre* (E–H,M–P) distal retina at E16 and E17, as indicated. *Pax6* ^-^ area was delineated by staining for Pax6 protein (E,M) on adjacent sections (dotted line in E–H,M,N). Expression of Ccnd2 (B,F) Ccnd3 (C,G), P57^Kip2^ (D,H) and P27^Kip1^ (green in J–L,N–P) is upregulated in most of the *Pax6* ^-^ RPCs of *Pax6* ^*loxP/loxP*^ ;α*-Cre* retina. Ccnd1 and P27^Kip1^, which are expressed in different cells in the control (J) are coexpressed in *Pax6* ^-^ RPCs (N). For the control and Pax6-deficient cells, P27^Kip1^-expressing cells are negative for Ki67 (red in K,O) and BrdU (red in L, P). Abbreviations: GCL, ganglion cell layer; NBL, neuroblastic layer. Scale bar in A is 100 µm.

Our microarray analysis revealed upregulation of both *CyclinD2* (*Ccnd2*) and the CKI *P57* ^*Kip2*^ in *Pax6* ^*loxP/loxP*^;α*-Cre* OC compared to controls (Table S1). We therefore monitored the expression of these and other cell-cycle factors in control and *Pax6* ^*loxP/loxP*^;α*-Cre* OCs during retinogenesis (Figure 4, Figure S4). In the control embryonic retina, Pax6 was detected in proliferating RPCs in the NBL as well as in differentiating neurons located in the inner nuclear layer (INL) and ganglion cell layer (GCL, Figure 4A,I). At these stages, Ccnd2, Ccnd3 and P57^Kip2^ were rarely detected in the RPCs (Figure 4B–D), whereas Ccnd1 and P27^Kip1^ were prominent in the NBL (Figure 4J [42] [43]). In contrast, we detected ectopic expression of Ccnd2, Ccnd3 and P57^Kip2^ (Figure 4F–H), as well as elevated expression of Ccnd1 and P27^Kip1^ (Figure 4N), within the Pax6-deficient RPCs, identified by monitoring loss of Pax6 protein on adjacent sections (Figure 4E,M). Moreover, while in the control retina Ccnd1^+^ and P27^Kip1+^ cells are mutually exclusive [43] (Figure 4J, inset), these factors were coexpressed in the *Pax6* ^*loxP/loxP*^;α*-Cre* retina (Figure 4N, inset). Despite the misexpression of Ccnd1, the Pax6^-^P27 ^Kip1+^ Ccnd1^+^ mutant cells were negative for Ki67 and did not incorporate BrdU (Figure 4O,P), similar to *P27* ^*Kip1+*^ cells in the control retina (Figure 4K,L). Thus, aberrant expression of Ccnd1 in these cells is not sufficient to maintain them in a proliferative state.

Elevated expression of Ccnd2 and P57^Kip2^ proteins was also detected in a small number of cells at E12.5, consistent with the E12 transcriptomic data (not shown). Interestingly, the microarray data for Ccnd1 indicated reduced transcript levels in the distal *Pax6* ^*loxP/loxP*^;α*-Cre* retina compared to controls (fold change -1.5). Nevertheless, IIF for Ccnd1 protein indicated maintained and even elevated protein levels (E13.5, E15.5, Figure S4G,N; E17.5, Figure 4N). We therefore monitored Ccnd1 transcript and protein at E12.5, E13.5 and E15.5 (not shown and Figure S4). At these stages, the normal RPCs co-express Pax6 and Ccnd1 (Figure S4A,B,I,J), while the postmitotic Crx^+^ photoreceptor precursors do not express either Pax6 or Ccnd1 (Figure S4C,D,K,L and [8]). In contrast, in the *Pax6*-mutant RPCs, we detected a reduction in Ccnd1 transcript from E13.5 which was prominent by E15.5 (Figure S4F,N). Interestingly, although Ccnd1 transcript was gradually reduced, Ccnd1 protein was abnormally retained as it was detected in the Pax6^-^Crx^+^ cells at E13.5 (Figure S4G,H) and in the neurogenic population (Pax6^-^Crx^-^ cells) at E15.5 and E17.5 (Figure S4O,P, Figure 4N). This finding suggests complex regulation of Ccnd1 by Pax6, as it is required for both normal levels of Ccnd1 transcription and regulation, and through posttranscriptional/translational mechanisms, of Ccnd1 protein levels.

Taken together, the *Pax6* ^-^ retinal precursors display elevated expression of both cell-cycle-promoting (*Ccnd1–3* and PCNA) and cell-cycle-inhibiting (*P27* ^*Kip1*^ and *P57* ^*Kip2*^) factors. These results indicate a major role for *Pax6* in controlling the events leading to timely cell-cycle exit of RPCs.

### TFs that Regulate RPC Proliferation and Cell Cycle Are Abnormally Expressed in Pax6^-^ Retina

The above results indicate a key role for *Pax6* in controlling cell-cycle parameters. We therefore focused our analysis on TFs whose expression was altered based on the microarray analysis and which have been previously shown to regulate RPC proliferation and thus could mediate *Pax6*’s role in the regulation of cell-cycle dynamics.

**Figure 5:**
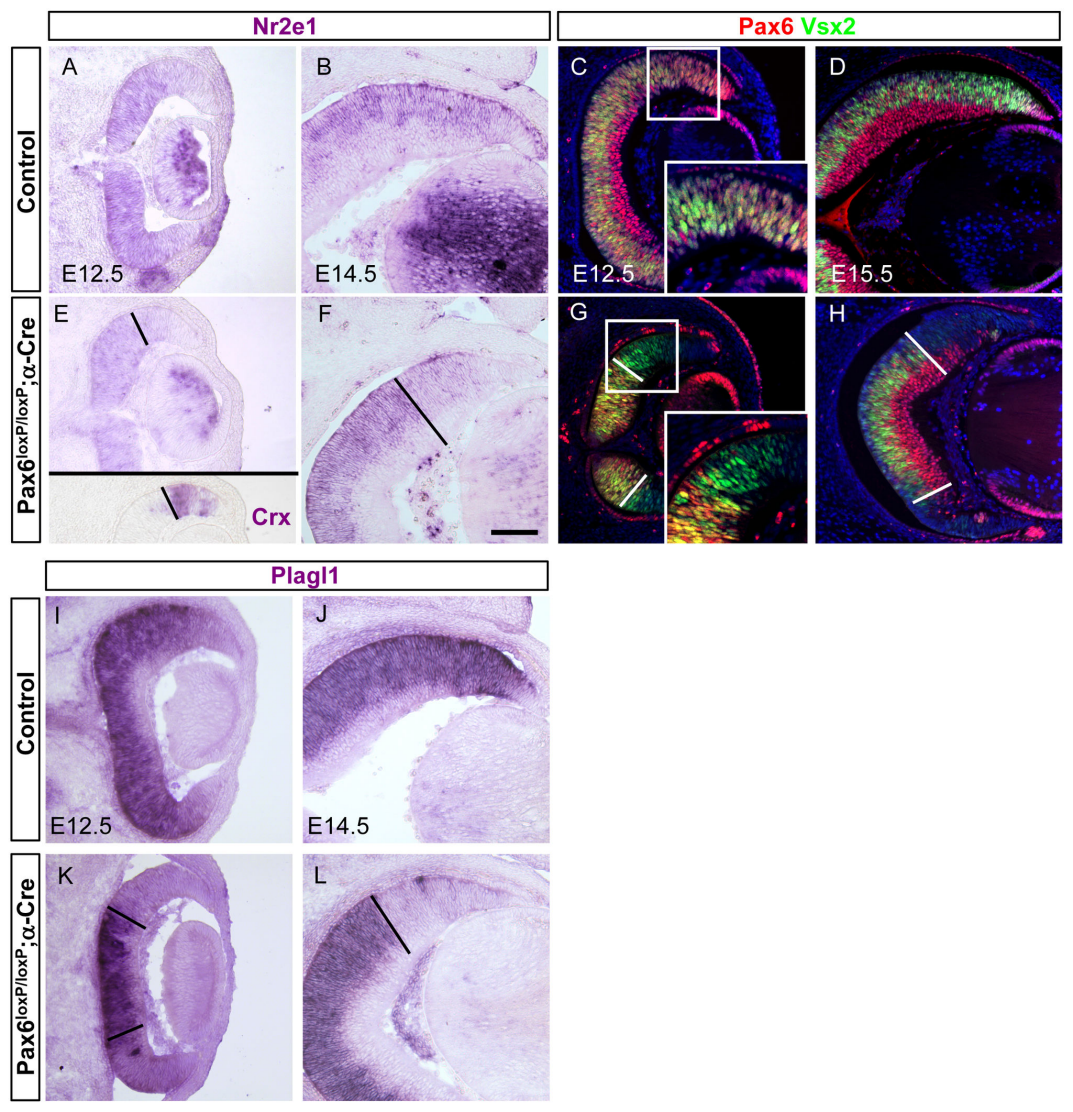
Abrogated expression of factors implicated in regulating RPC proliferation in *Pax6* ^*loxP/loxP*^ ;α*-Cre* retina. The expression pattern of different factors was monitored on sections from eyes of control (A-D,I,J) and *Pax6* ^*loxP/loxP*^ ;α*-Cre* (E-H,K,L) mice. Nr2e1 (E12.5 A,E; E14.5 B,F) and Plagl1 (E12.5 I,K; E14.5 J,L) were detected using ISH while Vsx2 (green; E12.5 C,G; E15.5 D,H) Pax6 (red; E12.5 C,G; E15.5 D,H) were detected by IIF analysis. Pax6 (red in C,D,G,H and not shown) and Crx (inset in E and not shown) expression were used to delineate the different RPC populations in the *Pax6^loxP/loxP^*;α*-Cre* retina (marked with dotted line in E-H,K,L). Scale bar in F is 100 µm.

The homeodomain TF *Vsx2* and the orphan nuclear receptor *Nr2e1* (*tailless*, *Tlx*) have been previously reported to promote RPC proliferation [43-45]. Loss-of-function mutations in both genes result in increased expression of *P27* ^*Kip1*^, reduced expression of *Ccnd1* and a hypoplastic retina [43-45]. Expression of these TFs was found to be reduced in the *Pax6* ^*loxP/loxP*^;α*-Cre* retina based on the microarray analysis (fold change -1.9 and -2.18 for *Vsx2* and *Nr2e1*, respectively). We therefore examined the dynamics of their expression following *Pax6* deletion in situ. Both *Nr2e1* and *Vsx2* expression was detected in most proliferating RPCs in the NBL of control retinas (Figure 5A–D); their expression was slightly reduced at E12.5 in *Pax6* ^*loxP/loxP*^;α*-Cre* retinas (Figure 5E,G; Pax6 mutant region identified by IIF on adjacent section) and it was virtually abolished by E14.5 in *Pax6* ^*loxP/loxP*^;α*-Cre* mutants (Figure 5F,H). A reduction in the expression of these neuronal progenitor genes could mediate the reduced proliferation of the *Pax6*-deficient RPCs.

The maternally imprinted tumor suppressor TF *Plagl1* (*Zac1*) is a zinc-finger protein that can act as both a transcriptional activator and repressor [46]. Loss of *Plagl1* results in a hypercellular retina containing an additional INL composed of amacrine cells [15]. As previously described, *Plagl1* was widely expressed throughout the NBL in control mice (Figure 5I,J and [15]) but its expression was lost from all *Pax6* ^-^ RPCs (identified by IIF, not shown) in the distal retina of *Pax6* ^*loxP/loxP*^;α*-Cre* as early as E12.5 (Figure 5K,L).

These results reveal that *Pax6* is required for the expression of TFs known to promote (*Vsx2* and *Nr2e1*) and restrict (*Plagl1*) RPC proliferation and thus *Pax6* loss in RPCs results in a dramatic alteration in cell-cycle dynamics (see discussion).

Figure S5A–D, I–L [48]). The microarray analysis showed reduced expression of *Hes5* and *Dll1* at E12. Indeed, expression of the Notch-pathway genes *Notch1*, *Dll1* and *Hes5* was reduced in the *Pax6* ^*-*^ *Crx* ^*+*^ population of E13.5 *Pax6* ^*loxP/loxP*^;α*-Cre* retina (Figure S5E–H). However, at later stages of development, when the neurogenic population (*Pax6* ^*-*^;*Crx* ^-^) is prevalent, the expression of *Notch1* and *Dll1* was similar to their expression levels in the control OC (E16.5, Figure S5N,O), although the expression of *Hes5* was reduced in some, but not all of the Pax6-mutant OC, probably reflecting a reduction in RPCs (Figure S5P). These findings suggest that Pax6 may be required for the Notch pathway in the most distal OC (*Pax6* ^*-*^ *Crx* ^*+*^), but its activity is not essential for maintaining the expression of Notch-signaling components in the more central RPCs. Therefore, the reduced proliferation of the *Pax6* ^*-*^;*Crx* ^-^ neurogenic RPCs is probably not due to altered activity of Notch signaling.

HH signaling is required to maintain normal proliferation of RPCs, as loss of HH signaling results in precocious cell-cycle exit and depletion of the progenitor pool [51,52], whereas overactivation of the HH pathway results in increased proliferation and retinal hyperplasia [53]. HH signaling has also been shown to regulate the expression of various cell-cycle-related factors such as *Ccnd1*, *P57* ^*Kip2*^ and *Mycn* [51-54]. The microarray analysis conducted on E12 control and *Pax6* ^*loxP/loxP*^;α*-Cre* retinas yielded no differential expression of HH pathway-related factors, possibly because at this stage *Shh* components are not prominent in the peripheral OC in regions of α*-Cre* activity [55].

**Figure 6:**
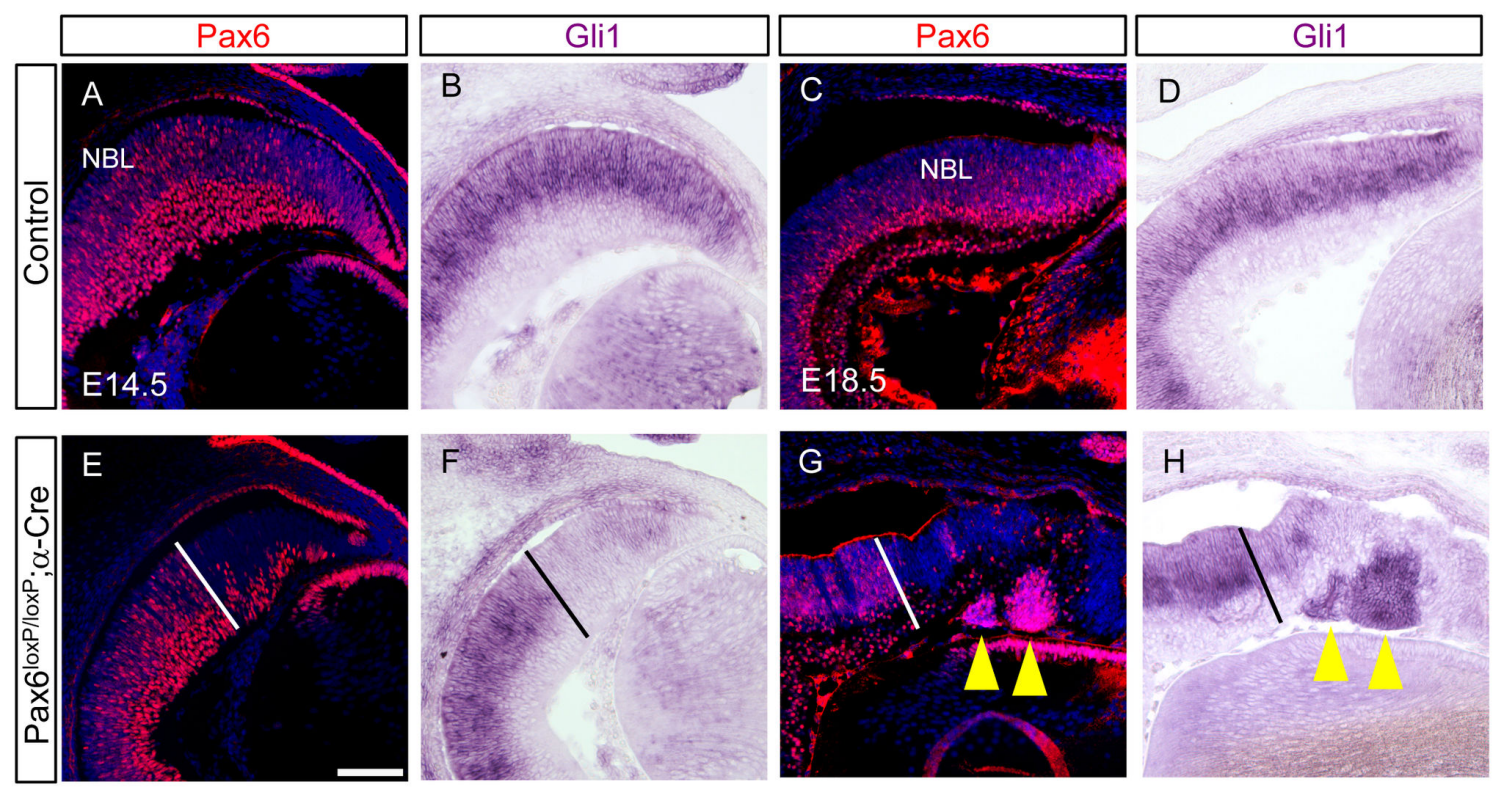
Hedgehog signaling is disrupted in *Pax6* ^*loxP/loxP*^ ;α*-Cre* retina. Expression of Gli1 was detected using ISH (B,D,F,H) and Pax6 protein was detected by IIF on adjacent sections (A,C,E,G) for control (A-D) and *Pax6* ^*loxP/loxP*^ ;α*-Cre* (E-H) OC. Gli1 expression was abrogated in all *Pax6* ^-^ RPCs (F,H) compared to controls (B,D) at both E14.5 (B,F) and E18.5 (D,H). Pax6^-^ area was determined by antibody labeling on adjacent sections and marked by dotted line (E-H). Scale bar in B is 100 µm.

To test for possible alterations in HH signaling, we characterized *Gli1* expression at various developmental stages, as *Gli1* is both an effector and a target of the HH pathway (Figure 6B,D,F,H [56]). IIF analysis for Pax6 was performed on adjacent sections to determine the Pax6 mutant area (Figure 6A,C,E,G). As previously reported, extensive *Gli1* expression was detected in the NBL of both E14.5, E1.5.5 and E18.5 control retinas (Figure 6B,D, Figure S6A). However, in the distal retina of *Pax6* ^*loxP/loxP*^ *;* α*-Cre*, *Gli1* expression was undetectable throughout retinogenesis (E14.5, E18.5, Figure 6F,H, Figure S6D) suggesting HH-signaling arrest in *Pax6* ^-^ RPCs. The expression of *Gli2* and *Gli3*, which participate in HH signaling but are not HH-signaling targets [57], was unaltered in *Pax6* ^*loxP/loxP*^ *; α-Cre* compared to controls (Figure S6) and thus these mediators of the HH pathway are maintained despite the loss of *Pax6*. To further determine whether *Pax6* is required cell autonomously for HH signaling, we examined *Gli1* expression in RPCs that escaped *Cre*-mediated *Pax6* deletion. This occurred because the α*-Cre* transgene is occasionally inactive in some RPCs resulting in patches of nonrecombinant cells surrounded by recombinant ones. In the distal OC of the *Pax6* ^*loxP/loxP*^ *; α-Cre* embryos, these patches of *Pax6*-expressing RPCs form rosette-like structures (Figure 6G,H, yellow arrowheads). These rosettes share the same environment as recombinant *Pax6* ^-^ RPCs, yet extensive *Gli1* expression was detected in the *Pax6* ^+^ rosettes (Figure 6H, yellow arrowheads). These results suggest that *Pax6* plays a cell-autonomous role within the RPCs for the normal activation of HH signaling.

## Discussion

### *Pax6*-Deficient RPCs Exhibit a Unique Cell-Cycle Phenotype

Previous studies have substantiated the requirement for *Pax6* in establishing the neuronal progenitor pool during development of the vertebrate nervous system. Reduced proliferation was documented following knockdown of *Pax6* in the embryonic chick retina and spinal cord [12,58]. Similarly, reduced BrdU incorporation was detected in the diencephalon and altered proliferation was observed in the dorsal telencephalon of *Pax6*-knockout mice [11,59,60]. Corresponding with alterations in the cell cycle, the expression of cell-cycle factors was noted in various *Pax6* mutants, including increased expression of *Ccnd1* in the *Pax6*-deficient cortex during early corticogenesis, and elevation of both *Ccnd2* and *P57* ^*Kip2*^ during late stages of cortical development [40]. Increased levels of *Ccnd1* were reported in zebrafish embryos following knockdown of *Pax6* proteins [61].

**Figure 7:**
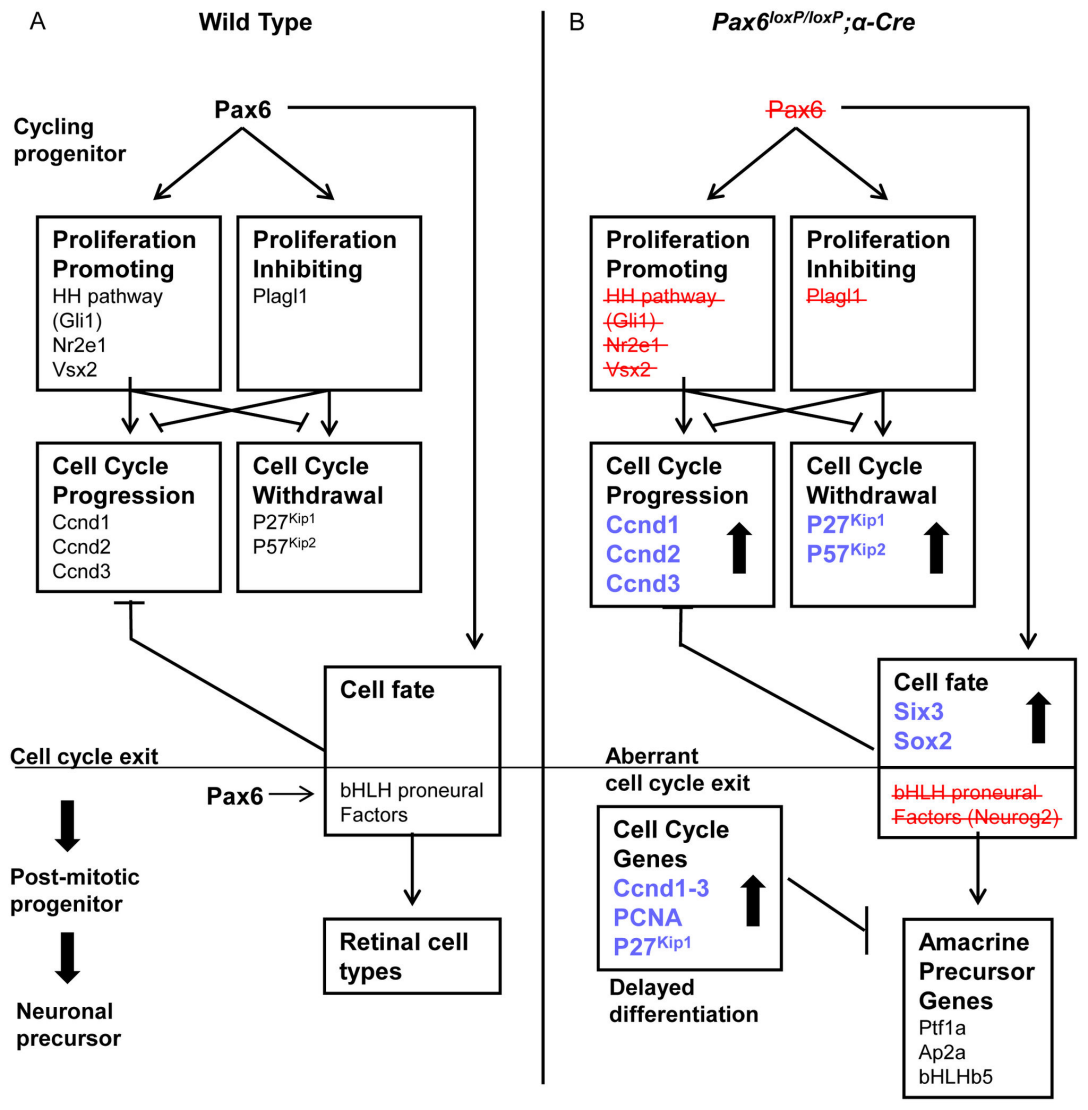
Scheme depicting *Pax6* roles during the transition from proliferating retinal progenitor to differentiating retinal neuron. (A) In normal cycling retinal progenitors, *Pax6* regulates the balance between proliferation promoting (i.e. *Nr2e1*, *Vsx2*, Hedgehog (HH) signaling) and inhibiting factors (i.e. *Plagl1*). These in turn regulate the expression of genes which induce either progression of (i.e. *Ccnd1–3*) or withdrawal (*P27* ^*Kip1*^, *P57* ^*Kip2*^) from the cell cycle. It is also required for the expression of bHLH proneural factors (*Neurog2*, *Atoh7*, and *Ascl1*) presumed to inhibit cell-cycle factors as well as promote specific retinal lineages. (B) *Pax6* loss from RPCs results in aberrant cell-cycle exit as *Ccnd1–3*, as well as *P27* ^*Kip1*^ and *P57* ^*Kip2*^, are elevated and several cell-fate determination factors show reduced (bHLH proneural factors) or increased (*Six3*, *Sox2*) expression. The combined outcome of these alterations is delayed differentiation of the *Pax6*-deficient cells to only one subclass of retinal interneurons.

In the current study, we observed that in *Pax6*-deficient retina there is a reduced number of cells in the S phase, accompanied by a significant and progressive increase in the number of cells exhibiting cell-cycle abnormalities. These were manifested in the elevated expression of cell-cycle-progression factors (*Ccnd1*, *Ccnd2*, *Ccnd3*) and cell-cycle-withdrawal factors (CKIs: *P27* ^*Kip1*^ and *P57* ^*Kip2*^, Figure 4, Figure 7). Despite their opposing functions, increased expression of both types of cell-cycle factors can contribute to delayed differentiation of the *Pax6*-deficient neuronal precursors. This is supported by the finding that overexpression of *Ccnd1* in photoreceptor precursors delays their differentiation [62]. Beyond the CNS, *Ccnd1* was further shown to inhibit adipocyte and myoblast differentiation [63-65]. In addition, while the increase in *P27* ^*Kip1*^ and *P57* ^*Kip2*^ promotes cell-cycle exit, it is now recognized that this increase is not sufficient to induce cell differentiation, which depends on additional cues [66-68]. Moreover, *P27* ^*Kip1*^ has been recently shown to contain CDK-independent functions that inhibit the differentiation of postmitotic cells. These oncogenic activities of *P27* ^*Kip1*^ were recognized in mice expressing a mutant isoform of *P27* ^*Kip1*^ which is unable to bind cyclin–CDK. In these mice, in contrast to *P27* ^*Kip1*^-knockout mice, ectopic proliferation and differentiation arrest were detected in the developing retina [69]. It is therefore likely that the elevation of both cell-cycle-promoting and inhibiting factors in the *Pax6*-deficient RPCs contributes to their delayed differentiation to retinal neurons.

### Pax6 Regulates a Transcriptional Network Capable of both Promoting and Inhibiting the Transition of Proliferating RPCs into Postmitotic Precursors

A few studies have suggested the direct involvement of *Pax6* in cell-cycle mechanisms by regulating cell-cycle-related factors such as *P27* ^*Kip1*^ [39] and interacting with *Rb* [70]. Other findings propose roles for *Pax6* in cell division, including sister-chromatid separation [71], interkinetic nuclear movement and determination of the orientation of cell division [72,73]. . While these *Pax6* functions may have some effect on the cell-cycle kinetics of *Pax6* ^-^ RPCs, it is unlikely that they account for the profound alterations in the expression of multiple cell-cycle factors observed in the *Pax6* ^*loxP/loxP*^;α*-Cre* retina.

Through the characterization of cell-cycle and cell-differentiation dynamics together with transcriptional changes in *Pax6* ^-^ RPCs, the current study reveals that *Pax6* functions upstream of multiple factors, each of which is an important determinant in the regulation of RPC proliferation. We observed reduced expression of several factors that are required for proliferation of retinal progenitors during early stages of retinogenesis: *NR2e1*, *Vsx2* and HH signaling (Figure 7). The loss of each of these components is expected to result in reduced proliferation, as observed in the *Pax6*-mutant retina.

*Nr2e1* is an essential intrinsic regulator of neural stem cells during embryonic development and in adult neurogenesis [74,75]. *Nr2e1* has been found to play a key role during multiple stages of eye development. Functional studies in frog embryos suggest that the *Nr2e1* homolog *Xtll* is an eye-field gene and required for normal eye formation [76,77]. In murine embryos, *Nr2e1* seems to be dispensable for specification of retinal cell types but consistent with its activities in the cortex, it is required for normal proliferation of RPCs through activation of *Ccnd1* and inhibition of *P27* ^*Kip1*^ [44]. Similar to *Nr2e1*, *Vsx2* is expressed in RPCs prior to the onset of retinal neurogenesis. It is initially required at the OV stage for ocular neuroectoderm patterning to the retinal pigmented epithelium (RPE) and retinal lineages, while at the OC stage it is primarily involved in promoting RPC proliferation [78,79]. Though *Vsx2* expression is maintained during early stages in the optic rudiment of *Pax6*-knockout mice [30], its expression was lost at later stages of development in both the systemic and *Pax6* ^*loxP/loxP*^;*α-cre* OC (Figure 5 and [30]). From this we can conclude that *Pax6* is required for *Vsx2* maintenance in RPCs but not for initiating its expression in the OV. *Vsx2*-knockout retinas exhibit reduced expression of *Ccnd1* and elevated levels of *P27* ^*Kip1*^, as well as cryptic coexpression of both *Ccnd1* and *P27* ^*Kip1*^. The inhibition of *P27* ^*Kip1*^ by *Vsx2* is thought to mediate its activity in RPCs, as proliferation is recovered in *P27* ^*Kip1*^;*Vsx2*-double-knockout mice [43]. The third positive regulator of proliferation that was lost in the *Pax6*-mutant RPCs is HH signaling, based on the loss of the target *Gli1* [80]. HH is an established mitogen in the developing CNS, including the retina [51,52,81]. The deletion of Smoothened, an essential mediator of this signal-transduction pathway, from RPCs resulted in progenitor-pool depletion due to cell-cycle aberrations which included reduced expression of *Ccnd1* and elevated expression of *P27* ^*Kip1*^ [51,52].

Considering the proliferation-promoting roles of *Nr2e1*, *Vsx2* and HH in RPCs, it is expected that combined reduction of these factors following *Pax6* loss will result in the reduced proliferation observed in the *Pax6* ^*loxP/loxP*^;α*-Cre* retina (Figure 7). Yet, in the *Pax6*-deficient RPCs, the reduced proliferation was accompanied by elevated expression of several cell-cycle-promoting factors: *Ccnd1*, *Ccnd2* and *Ccnd3*. This elevated expression could be due to altered expression of factors which normally inhibit cell-cycle progression (Figure 7). Among these is the tumor suppressor *Plagl1* [46]. Plagl1 is expressed from early stages of retinogenesis in RPCs, with higher levels in the distal OC and reduced levels in the central more mature RPCs [15]. Analysis of *Plagl1*-knockout mice revealed increased RPC proliferation and an increase in the number of *Ccnd1*-expressing cells [15]. In addition to loss of *Plagl1*, we detected increased expression of several progenitor factors in the *Pax6*-deficient RPCs such as *Six3* and *Sox2* (Figure 5, Figure S7). The increased expression of these progenitor genes combined with the loss of *Plagl1* probably contributes to the unique cell-cycle phenotype observed in the *Pax6*-mutant retina.

The distinctive molecular phenotype of *Pax6*-deficient RPCs suggests that *Pax6* simultaneously controls a number of genes which function during the transition of RPCs to differentiating precursors. Consistent with our findings, recent high-throughput analyses of *Pax6* function in the developing cortex, conducted using chromatin immunoprecipitation and transcriptomic profiling of both *Pax6* loss- and gain-of-function transgenic models, suggest a complex gene network mediating *Pax6* activity in cell proliferation and differentiation during cortical development [41]. The analysis suggested that *Pax6* can simultaneously regulate the expression of factors which promote cell proliferation and self-renewal (*CDK4* and *Hmga2* [82]) and cell-cycle inhibitors (tumor suppressor *Pten* [83]). Collectively, these observations suggest that *Pax6* controls several key TFs and signaling pathways, some with opposing roles, and that the phenotype observed in *Pax6* ^*loxP/loxP*^;α*-Cre* retinas is due to the combined disruption of several pathways.

The seemingly opposing activities of *Pax6* in RPCs may reflect dosage- and context-dependent activity of this TF in the heterogeneous population of progenitors. Indeed, the levels of *Pax6* vary according to cell-cycle stage; cells in the G1 and S phase express low levels of *Pax6*, whereas cells in G2 and M may display either low levels, high levels or no *Pax6* expression at all [58]. An additional level of complexity in deciphering the mechanism of *Pax6* activity is that this TF also regulates the expression of differentiation factors, particularly that of bHLH proneural factors *Ato7* [84,85], *Neurog2* [86] and *Ascl1* [6-8]. These differentiation-promoting TFs are thought to directly inhibit cell-cycle-promoting factors as was demonstrated for *Neurog2* [4]), and thus their combined loss may contribute not only to the limited differentiation potential of *Pax6*-deficient RPCs but also to the persistent expression of *Ccnd1* in the *Pax6*-deficient precursors. Finally, in addition to regulating multiple downstream TFs, it is now established that Pax6 controls the expression of microRNAs [87]. In future studies, microRNAs should be considered important mediators of Pax6 activity in posttranscriptionally controlling the expression levels of multiple genes simultaneously.

### *Pax6* Regulates Multiple Factors that Affect Retinal Cell-Fate Specification

In addition to reduced proliferation, the *Pax6*-deficient RPCs are limited in their differentiation potential as they only give rise to subclasses of GABAergic amacrine, while other retinal lineages fail to differentiate [6]. This limited multipotency was previously attributed to abrogated expression of the proneural bHLH proteins [6,8]. In the present study, we recognized additional perturbation in the *Pax6*-mutant OC that might account for the eventual acquisition of the amacrine fate (Figure S7). These changes included elevated expression of the amacrine-promoting factors Sox2 (Figure S7A,D) and Six3 (Figure S7B,E), as well as maintained expression of NeuroD1 in the NBL (Figure S7C,F, green). Ectopic expression of *Sox2* was shown to induce amacrine cells in the mouse retina [88] and similarly, overexpression of *Six3* together with *Neurod1* promoted amacrine cell genesis [89]. Furthermore, we detected reduced expression of *Plagl1* (Figure 5I-L). In addition to its role in regulating RPC proliferation, *Plagl1* functions in the regulation of amacrine cell number by activating a feedback mechanism by which amacrine cells can inhibit the formation of additional amacrines from RPCs [15]. Thus, loss of *Plagl1* in the *Pax6* ^*loxP/loxP*^;α*-Cre* retina might contribute to the formation of excessive numbers of amacrine cells. Collectively, these findings suggest that *Pax6* loss results in a unique transcription profile in the retinal precursors, which promotes the generation of amacrine interneurons. Interestingly, although these *Pax6*-mutant amacrine cells express GABA, their molecular phenotype was distinct from that of normal amacrine cells. In the *Pax6*-mutant retina, Sox2 protein was detected in many of the amacrine cells but only a small subset of these coexpressed Isl1 and only a few of these coexpressed choline acetyltransferase (ChAT; Figure S8E–H and [88]). In contrast, in the normal retina, the expression of Sox2 in amacrine cells is restricted to Isl1- and ChAT-expressing cells (Figure S8A–D). These findings suggest that while Pax6 is dispensable for the generation of GABAergic interneurons, it is required for normal differentiation of the amacrine cell types.

Future studies, employing *Cre* lines which are expressed in late progenitors and in postmitotic precursors, are required to determine the roles of *Pax6* in the generation of the late-born amacrine cell types (glycinergic and non-GABAergic-non-glycinergic [90,91]) as well as in the differentiation of the postmitotic amacrine precursors.

## Supporting Information

Figure S1 **Reduced expression of amacrine precursor markers in the *Pax6*^*loxP/loxP*^** ;**α-Cre retina.** Expression of amacrine specification and differentiation markers in control (A–D) and *Pax6* ^*loxP/loxP*^ ;α-Cre (E–H) OC. IIF was employed for the detection of Pax6 and VC1.1 (E15.5, green and red, respectively, in A,E) Ptf1a (E15.5, green in B,F), syntaxin and Ap2β (E16.5, red and green, respectively, C, G). BarHL2 (E16.5, D,H) was detected using ISH. The recombinant area in the *Pax6^loxP/loxP^*;*α-Cre* retina (marked with dotted line in E–H) was determined by monitoring Pax6 expression by IIF on an adjacent section (E,G adjacent to F,H respectively). Abbreviations: GCL, ganglion cell layer; NBL, neuroblastic layer. Scale bar in A is 100 μm for A,B,E,F. Scale bar in C is 100 µm for C,D ,G,H.
(TIF)

Figure S2 **Expression of PCNA and Ki67 does not overlap in a subset of *Pax6*^*-*^ RPCs.** A single pulse of BrdU was administered 24 h prior to sacrifice at E18.5 (A,B) or E15.5 (C,D). Sections of control (*Pax6^loxP/loxP^*;A,C) and *Pax6* ^*loxP/loxP*^ ;*α-Cre* (B,D) optic cup were double-stained by IIF with antibodies against BrdU (green in A–D) and either PCNA (red in A, B) or Ki67 (red in C,D). Pax6 expression was detected on adjacent sections and used to identify the recombination area in the *Pax6^loxP/loxP^*;*α-Cre* OC (dotted line in B,D,I–L, N). Coexpression of PCNA and Ki67 (red and green, respectively, in E–L) determined by IIF in control (E–H) and *Pax6* ^*loxP/loxP*^ ;α*-Cre* (I–L) retinas at E12.5 (E,I), E14.5 (F,J), E16,5 (G,K) and P0 (H,L). CyclinB1 expression detected by IIF at E13.5 in control and *Pax6^loxP/loxP^*;α*-Cre* OC (M,N). Abbreviation: NBL, neuroblastic layer. Scale bar in A is 100 µm.
(TIF)

Figure S3 **Gene ontology (GO) analysis of genes altered in *Pax6*^*loxP/loxP*^** ;**α*-Cre* compared to control RPCs**. Histogram depicting average significance of significantly enriched (*p*<0.05) GO terms as calculated using DAVID Bioinformatics Resources [32,33] clustered into functional and previously reported Pax6 functions.
(TIF)

Figure S4 **Aberrant expression of cell-cycle factors in the *Pax6* mutant OC.** IIF analysis for detection of Pax6 (A,E,I,M) ISH for detection of Ccnd1 transcript (B,F,J,N), IIF for Ccnd1 and Crx (red and green, respectively, C,D,G,H,K,L,O,P) in control (A–D, I–L) and *Pax6* ^*loxP/loxP*^ ;α*-Cre* (E–H, M–P) distal retina. Scale bar in A is 100 µm.
(TIF)

Figure S5 **Characterization of components of the Notch signaling pathway during retinogenesis in control and *Pax6*^*loxP/loxP*^** ;**α*-Cre* embryos**. Expression of Notch-pathway components at E13.5 (A-H) and E16.5 (I-P) in control (A-D, I-L) and *Pax6* ^*loxP/loxP*^ ;α*-Cre* (E-H, M-P) retinas. *Pax6* ^-^ area was delineated by staining for Pax6 protein on the same or adjacent sections (red in D-H,I,M; dotted line in E–H,M-P). Expression of Notch1 (B,F,J,N), Dll1 (C,G,K,O) and Hes5 (D,H,L,P) detected using fluorescent (A-H) or regular (I-P) ISH. Scale bar in A is 100 µm.
(TIF)

Figure S6 **Expression of Gli1 but not of Gli2 or Gli3 is decreased in the *Pax6*^*loxP/loxP*^** ;**α*-Cre* retina**. Expression of Gli1 (A,D) Gli2 (B,E) and Gli3 (C,F) in *Pax6* ^*loxP/loxP*^ control (A–C) and *Pax6* ^*loxP/loxP*^ ;α*-Cre* (D–F) optic cups as detected by ISH at E15.5. Scale bar in A is 100 µm.
(TIF)

Figure S7 **Altered expression of amacrine-differentiation-promoting and inhibiting factors in *Pax6*^*loxP/loxP*^** ;**α*-Cre* RPCs**. Control (A–C) and *Pax6* ^*loxP/loxP*^ ;*α-Cre* (D–F) embryonic retina labeled by IIF for Pax6 (E15, red, A,D,C,F) Sox2 (E15, green, A,D) and by ISH for detection of Six3 (E16, B, E), NeuroD1 (E15, green, C,F). Scale bar in A is 100 µm.
(TIF)

Figure S8 ***Pax6*^*-*^ amacrines display an abnormal molecular phenotype.** Control (A–D) and *Pax6* ^*loxP/loxP*^ ;*α-Cre* (E–H) P15 retina cholinergic amacrine labeled by IIF for Isl1 (red), choline acetyltransferase (ChAT, green in A,B,E,F) and Sox2 (green in C,D,G,H) Scale bar in A is 100 µm.
(TIF)

Table S1 **List of differentially expressed genes following Pax6 loss in Pax6^loxP/loxP^;α-Cre and control mice.**
(XLS)

Table S2 **List of differentially expressed genes found in the microarray analysis and validated in situ.**
(XLS)

Table S3 **List of primary antibodies used in this study.**
(PDF)
