# Supplementary figures and images for: Pax6 Is Required for Normal Cell-Cycle Exit and the Differentiation Kinetics of Retinal Progenitor Cells
Source: PLoS One. 2013 Sep 20;8(9):e76489. doi: 10.1371/journal.pone.0076489 (PMC3779171; doi:10.1371/journal.pone.0076489)

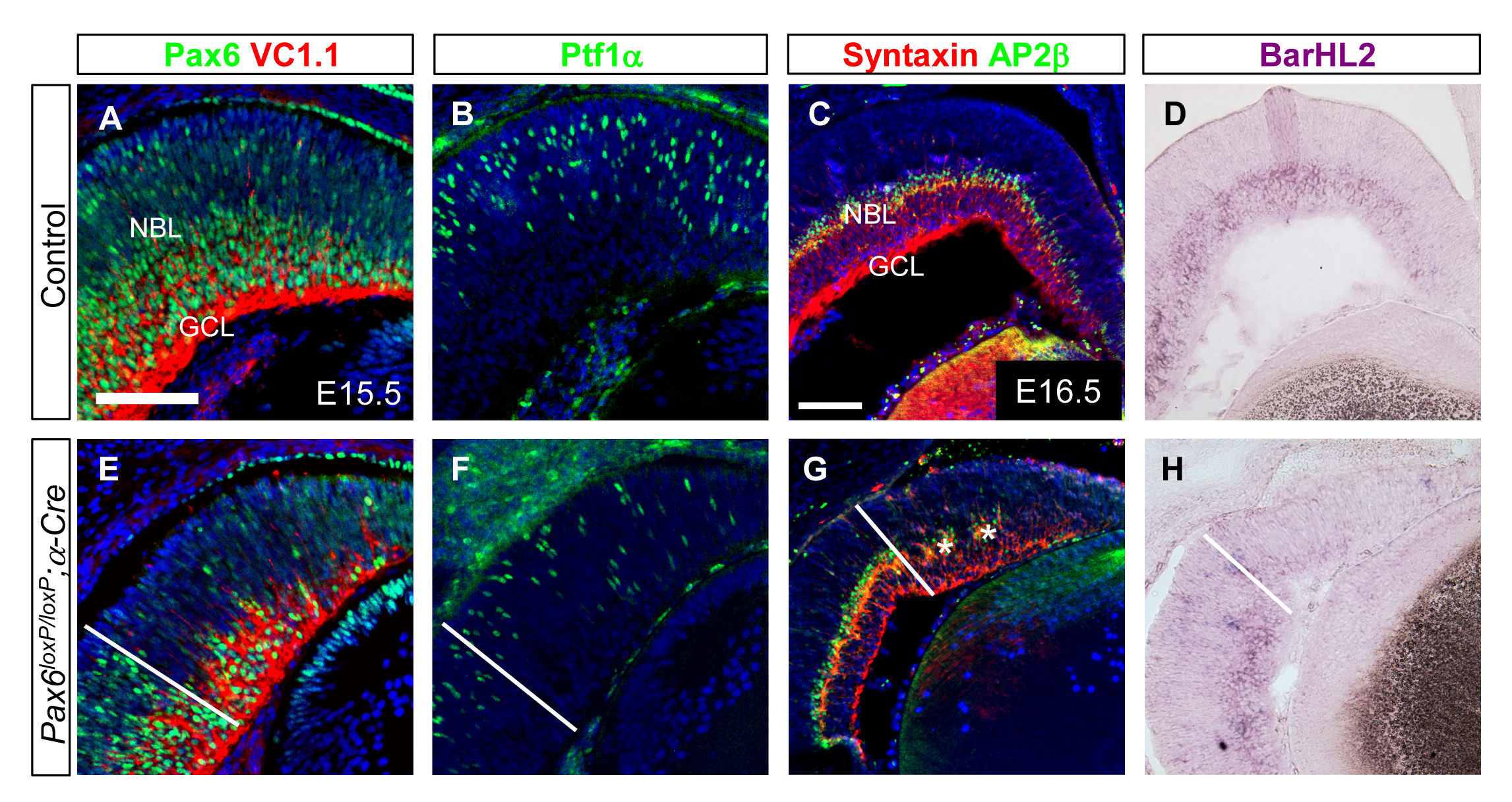

Supplement: Figure S1 — Reduced expression of amacrine precursor markers in the Pax6loxP/loxP ;α-Cre retina. Expression of amacrine specification and differentiation markers in control (A–D) and Pax6 loxP/loxP ;α-Cre (E–H) OC. IIF was employed for the detection of Pax6 and VC1.1 (E15.5, green and red, respectively, in A,E) Ptf1a (E15.5, green in B,F), syntaxin and Ap2β (E16.5, red and green, respectively, C, G). BarHL2 (E16.5, D,H) was detected using ISH. The recombinant area in the Pax6loxP/loxP;α-Cre retina (marked with dotted line in E–H) was determined by monitoring Pax6 expression by IIF on an adjacent section (E,G adjacent to F,H respectively). Abbreviations: GCL, ganglion cell layer; NBL, neuroblastic layer. Scale bar in A is 100 μm for A,B,E,F. Scale bar in C is 100 µm for C,D ,G,H. (TIF) [file pone.0076489.s001.tif]

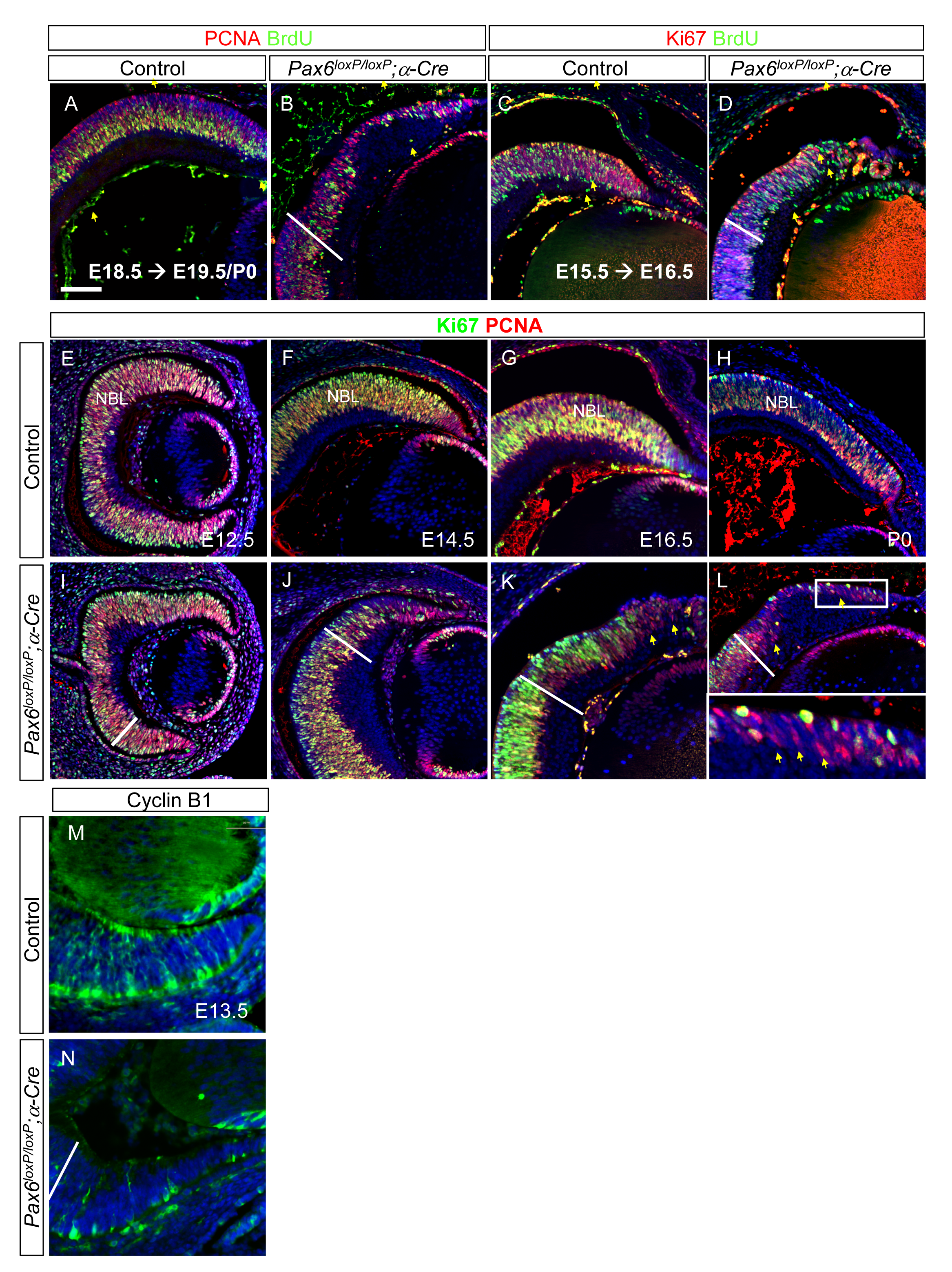

Supplement: Figure S2 — Expression of PCNA and Ki67 does not overlap in a subset of Pax6- RPCs. A single pulse of BrdU was administered 24 h prior to sacrifice at E18.5 (A,B) or E15.5 (C,D). Sections of control (Pax6loxP/loxP;A,C) and Pax6 loxP/loxP ;α-Cre (B,D) optic cup were double-stained by IIF with antibodies against BrdU (green in A–D) and either PCNA (red in A, B) or Ki67 (red in C,D). Pax6 expression was detected on adjacent sections and used to identify the recombination area in the Pax6loxP/loxP;α-Cre OC (dotted line in B,D,I–L, N). Coexpression of PCNA and Ki67 (red and green, respectively, in E–L) determined by IIF in control (E–H) and Pax6 loxP/loxP ;α-Cre (I–L) retinas at E12.5 (E,I), E14.5 (F,J), E16,5 (G,K) and P0 (H,L). CyclinB1 expression detected by IIF at E13.5 in control and Pax6loxP/loxP;α-Cre OC (M,N). Abbreviation: NBL, neuroblastic layer. Scale bar in A is 100 µm. (TIF) [file pone.0076489.s002.tif]

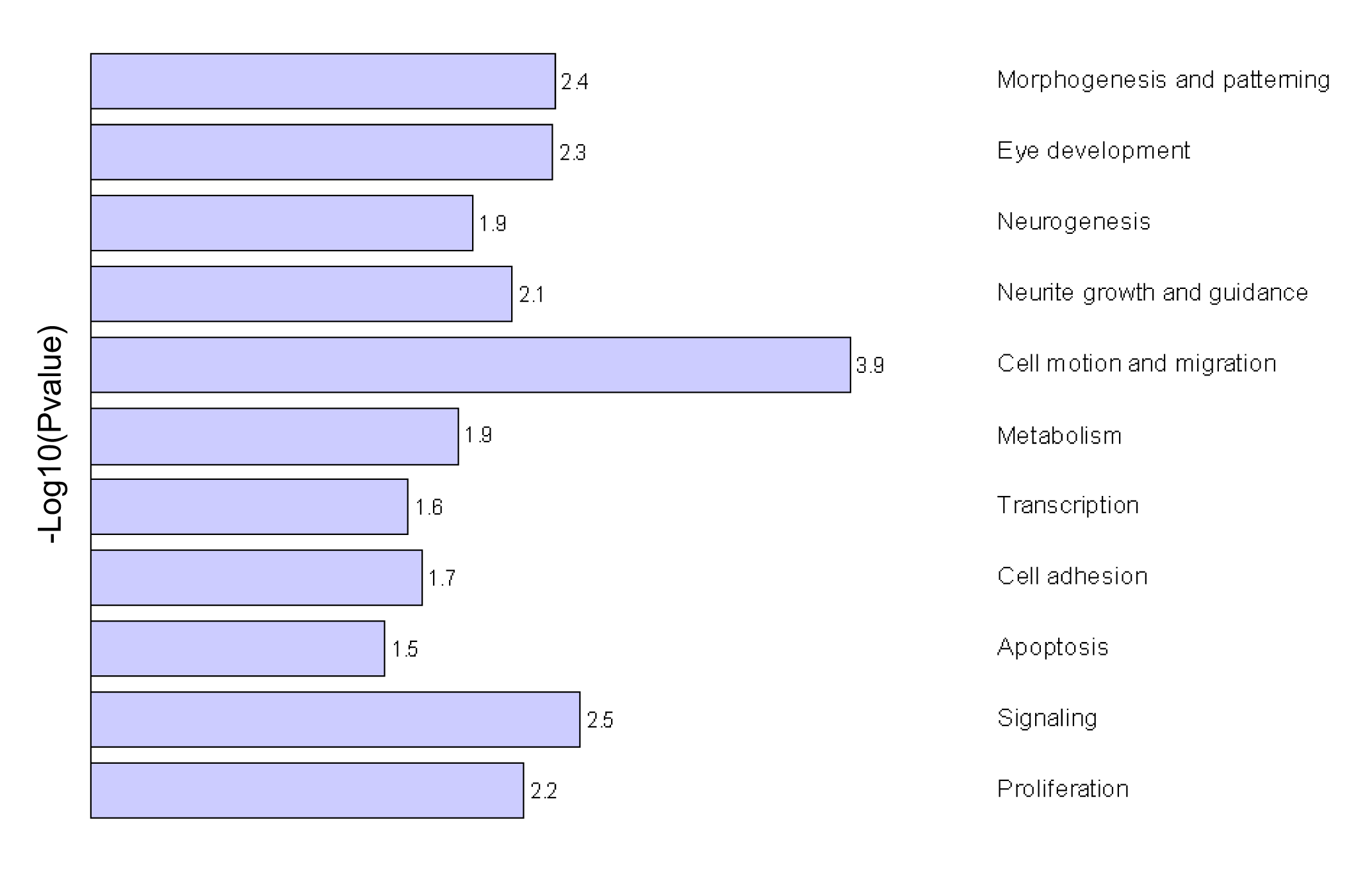

Supplement: Figure S3 — Gene ontology (GO) analysis of genes altered in Pax6loxP/loxP ;α-Cre compared to control RPCs. Histogram depicting average significance of significantly enriched (p<0.05) GO terms as calculated using DAVID Bioinformatics Resources [32,33] clustered into functional and previously reported Pax6 functions. (TIF) [file pone.0076489.s003.tif]

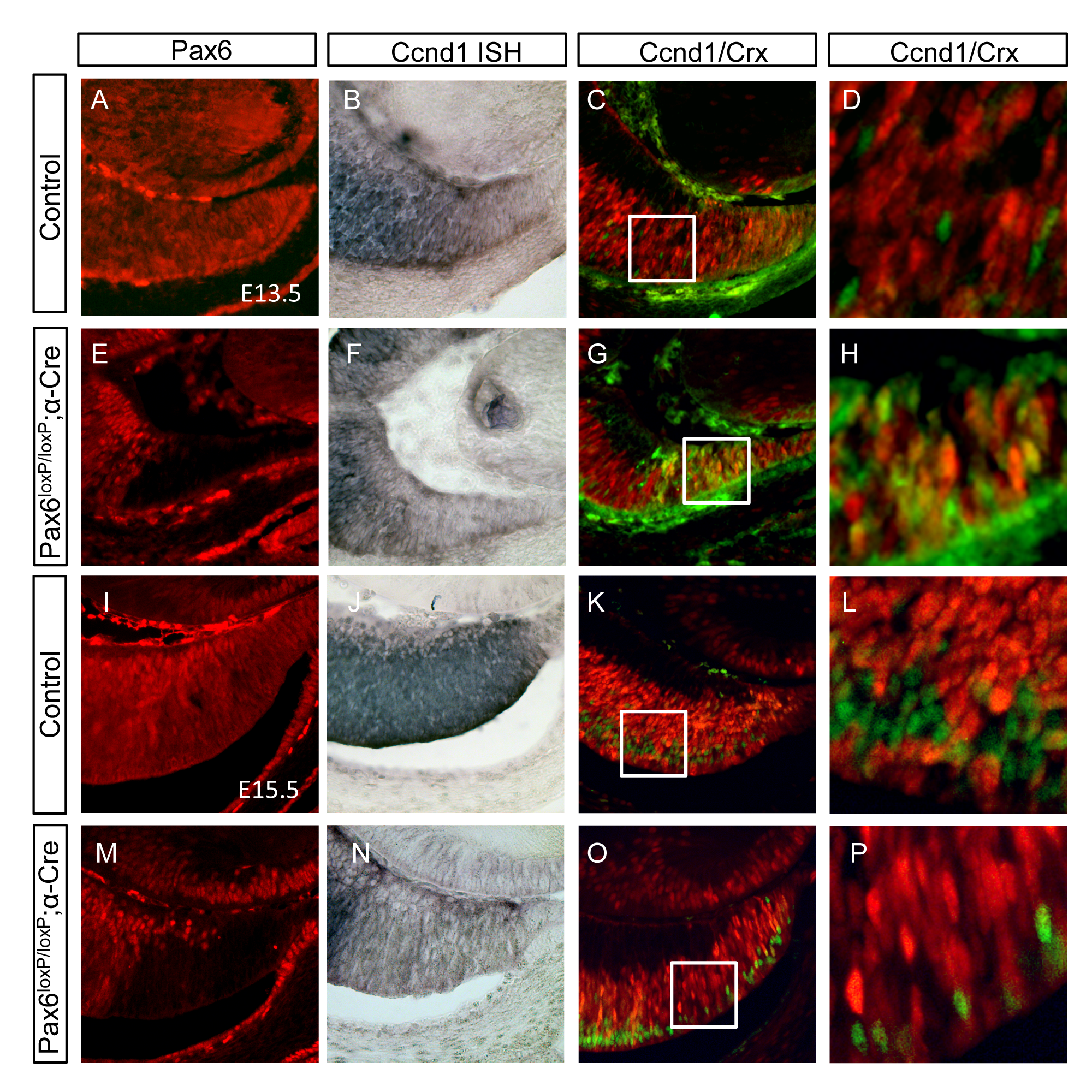

Supplement: Figure S4 — Aberrant expression of cell-cycle factors in the Pax6 mutant OC. IIF analysis for detection of Pax6 (A,E,I,M) ISH for detection of Ccnd1 transcript (B,F,J,N), IIF for Ccnd1 and Crx (red and green, respectively, C,D,G,H,K,L,O,P) in control (A–D, I–L) and Pax6 loxP/loxP ;α-Cre (E–H, M–P) distal retina. Scale bar in A is 100 µm. (TIF) [file pone.0076489.s004.tif]

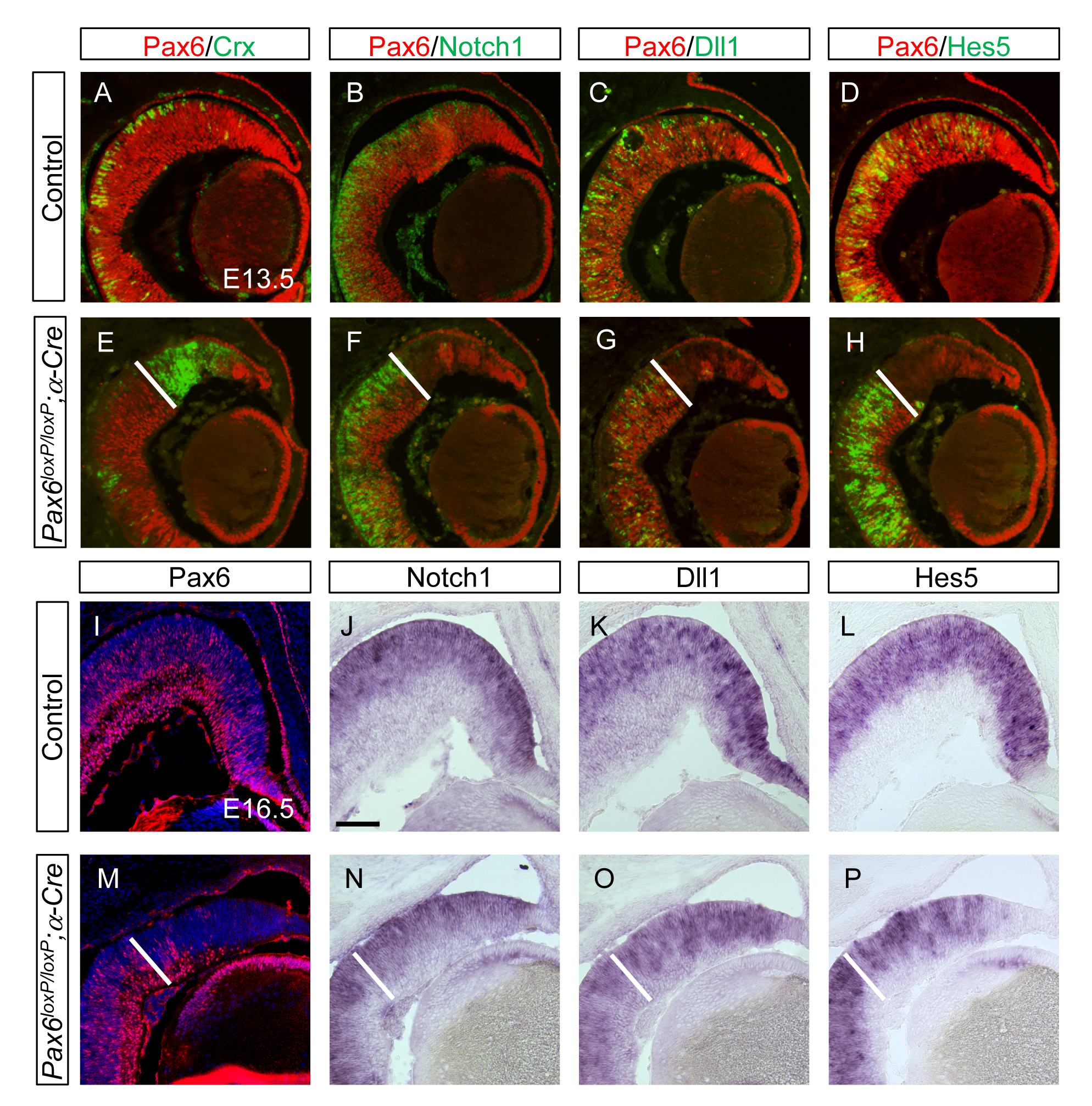

Supplement: Figure S5 — Characterization of components of the Notch signaling pathway during retinogenesis in control and Pax6loxP/loxP ;α-Cre embryos. Expression of Notch-pathway components at E13.5 (A-H) and E16.5 (I-P) in control (A-D, I-L) and Pax6 loxP/loxP ;α-Cre (E-H, M-P) retinas. Pax6 - area was delineated by staining for Pax6 protein on the same or adjacent sections (red in D-H,I,M; dotted line in E–H,M-P). Expression of Notch1 (B,F,J,N), Dll1 (C,G,K,O) and Hes5 (D,H,L,P) detected using fluorescent (A-H) or regular (I-P) ISH. Scale bar in A is 100 µm. (TIF) [file pone.0076489.s005.tif]

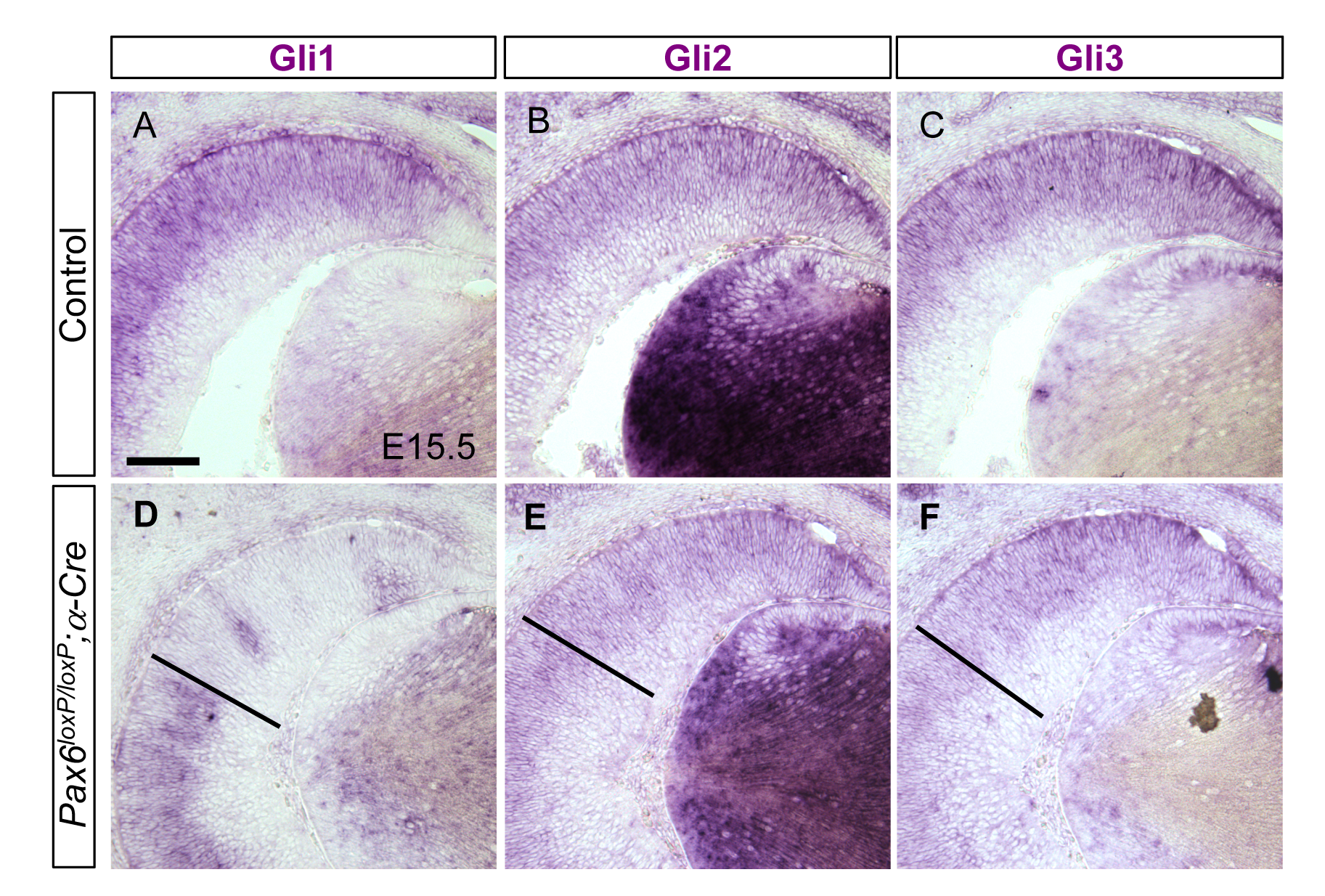

Supplement: Figure S6 — Expression of Gli1 but not of Gli2 or Gli3 is decreased in the Pax6loxP/loxP ;α-Cre retina. Expression of Gli1 (A,D) Gli2 (B,E) and Gli3 (C,F) in Pax6 loxP/loxP control (A–C) and Pax6 loxP/loxP ;α-Cre (D–F) optic cups as detected by ISH at E15.5. Scale bar in A is 100 µm. (TIF) [file pone.0076489.s006.tif]

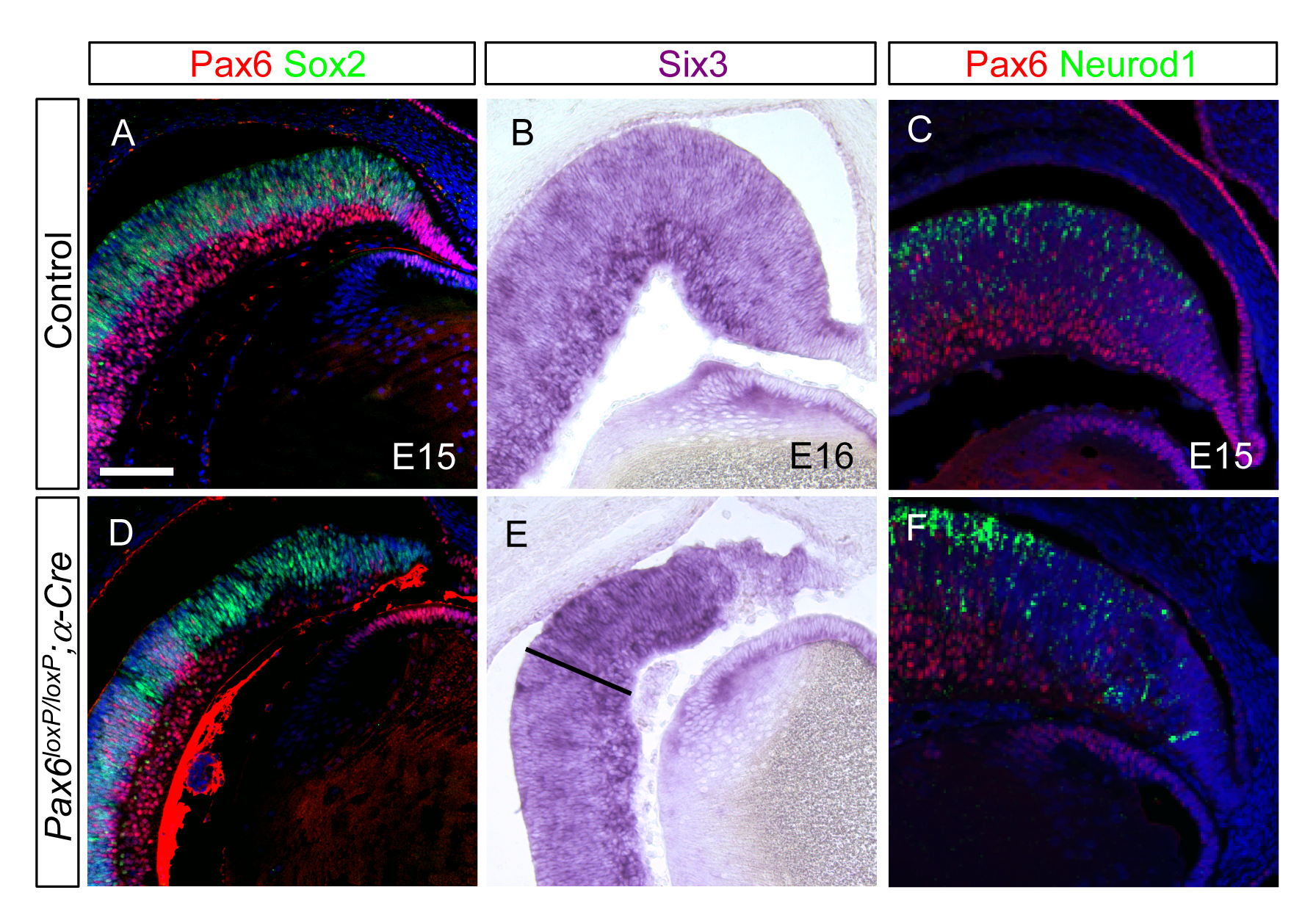

Supplement: Figure S7 — Altered expression of amacrine-differentiation-promoting and inhibiting factors in Pax6loxP/loxP ;α-Cre RPCs. Control (A–C) and Pax6 loxP/loxP ;α-Cre (D–F) embryonic retina labeled by IIF for Pax6 (E15, red, A,D,C,F) Sox2 (E15, green, A,D) and by ISH for detection of Six3 (E16, B, E), NeuroD1 (E15, green, C,F). Scale bar in A is 100 µm. (TIF) [file pone.0076489.s007.tif]

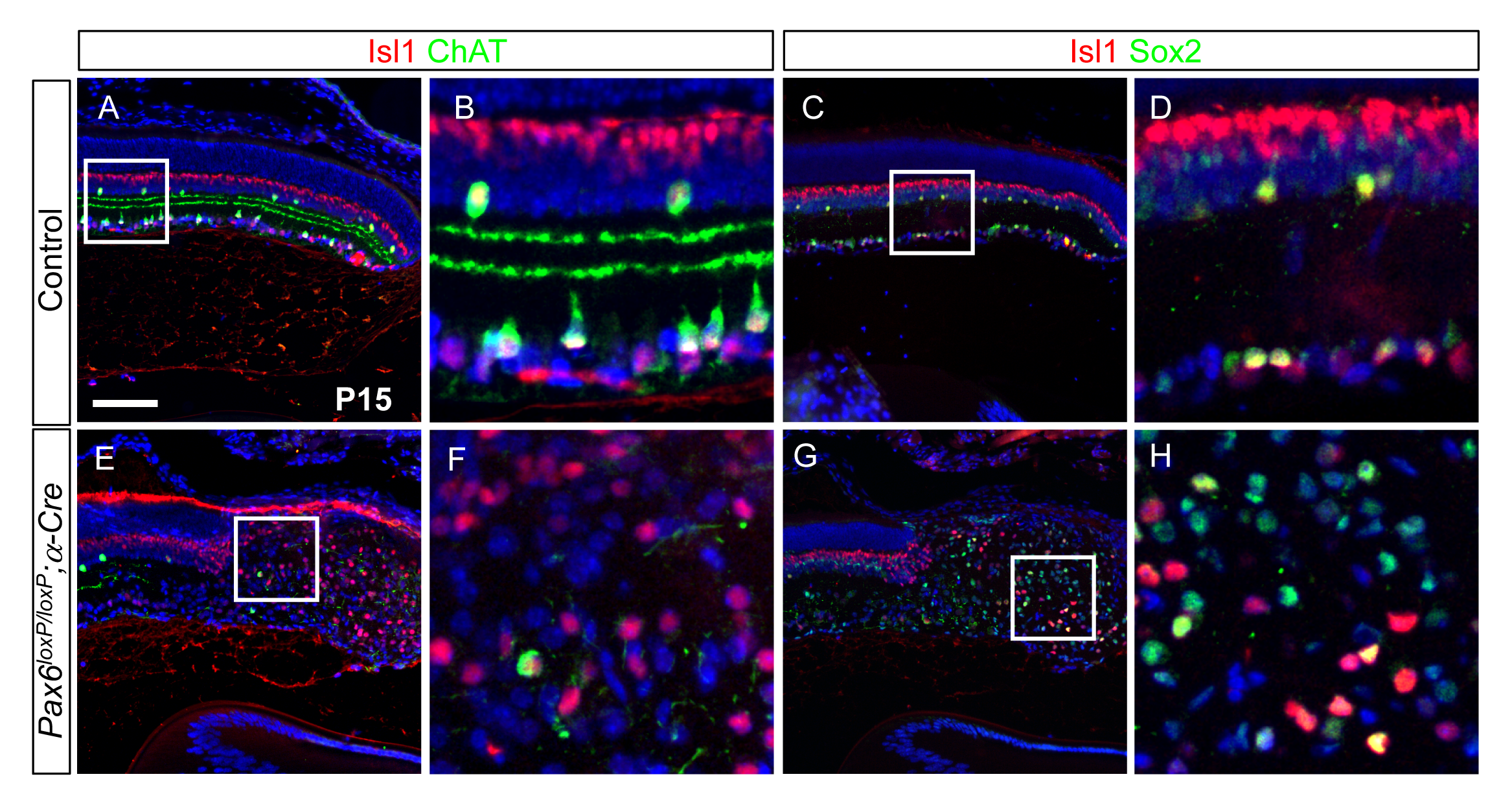

Supplement: Figure S8 — Pax6- amacrines display an abnormal molecular phenotype. Control (A–D) and Pax6 loxP/loxP ;α-Cre (E–H) P15 retina cholinergic amacrine labeled by IIF for Isl1 (red), choline acetyltransferase (ChAT, green in A,B,E,F) and Sox2 (green in C,D,G,H) Scale bar in A is 100 µm. (TIF) [file pone.0076489.s008.tif]
