# Supplementary material for: Pax6 Is Required for Normal Cell-Cycle Exit and the Differentiation Kinetics of Retinal Progenitor Cells
Source: PLoS One. 2013 Sep 20;8(9):e76489. doi: 10.1371/journal.pone.0076489 (PMC3779171; doi:10.1371/journal.pone.0076489)
Supplement: Table S3 — List of primary antibodies used in this study. (PDF) [file pone.0076489.s011.pdf]

Table S3. List of primary antibodies used in this study.

**Antigen**	**Source**	**Manufacturer**	**# Catalog**	**Dilution**
Pax6	Mouse	Santa Cruz	Sc-32766	1:25
Syntaxin	Mouse	Sigma	s0664	1:400
bHLHb5	Goat	Santa Cruz	sc-6045	1:300
AP2a	Mouse	Santa Cruz	sc-12726	1:50
Ptf1a	Rabbit	Kind gift of H. Edlund		1:500
GABA	Rabbit	Sigma	A2052	1:500
Sox2	Rabbit	Chemicon	AB5603	1:500
Vsx2	Sheep	Exalpha	X1180P	1:800
BrdU	Mouse	Chemicon	MAB3222	1:100
PCNA	Rabbit	Thermo Scientific	RB9055	1:100
Ki67	Rabbit	Thermo Scientific	RM9106	1:200
Ki67	Rat	Dako	M7249	1:100
Ccnd1	Rabbit	Thermo Scientific	Rm9104	1:250
Ccnd1	Mouse	Santa Cruz	Sc-450	1:50
Ccnd2	Rabbit	Santa Cruz	sc-593	1:50
Ccnd3	Rabbit	Santa Cruz	sc-182	1:50
$P27^{Kip1}$	Rabbit	Thermo Scientific	RB9019	1:100
$P57^{Kip2}$	Goat	Santa Cruz	sc-1039	1:50
